# Resensitizing β‐Lactams by Reprogramming Purine Metabolism in Small Colony Variant for Osteomyelitis Treatment

**DOI:** 10.1002/advs.202410781

**Published:** 2024-12-10

**Authors:** Tingwang Shi, Qiong Wu, Zesong Ruan, Zhiyuan Luo, Wenbo Wang, Zhao Guo, Yihong Ma, Xin Wang, Guangyu Chu, Han Lin, Min Ge, Yunfeng Chen

**Affiliations:** ^1^ Department of Orthopedic Surgery Shanghai Institute of Microsurgery on Extremities Shanghai Sixth People's Hospital Affiliated to Shanghai Jiao Tong University School of Medicine 600 Yishan Road Shanghai 200233 China; ^2^ Department of Laboratory Medicine Shanghai Sixth People's Hospital Affiliated to Shanghai Jiao Tong University School of Medicine 600 Yishan Road Shanghai 200233 China; ^3^ Department of Orthopedic Surgery Spine Lab The First Affiliated Hospital Zhejiang University School of Medicine Hangzhou 310003 China; ^4^ Joslin‐Beth Israel Deaconess Foot Center and The Rongxiang Xu MD Center for Regenerative Therapeutics Beth Israel Deaconess Medical Center Harvard Medical School Boston MA 02215 USA; ^5^ Shanghai Institute of Ceramics Chinese Academy of Sciences Research Unit of Nanocatalytic Medicine in Specific Therapy for Serious Disease Chinese Academy of Medical Sciences Shanghai 200050 China; ^6^ Department of Electrical and Electronic Engineering The University of Hong Kong Pokfulam Road Hong Kong 999077 China

**Keywords:** drug delivery, metabolic reprogramming, osteomyelitis, small colony variant, β‐Lactam resistance

## Abstract

Small colony variant (SCV) is strongly linked to antibiotic resistance and the persistence of osteomyelitis. However, the intrinsic phenotypic instability of SCV has hindered a thorough investigation of its pathogenic mechanisms. In this study, phenotypically stable SCV strains are successfully recovered from clinical specimens, characterized by elevated drug resistance and reduced immunogenicity. Multi‐omics analysis revealed that the acquired high drug resistance is associated with altered flux in the purine metabolism pathway, attributable to mutations in the hypoxanthine phosphoribosyltransferase (*hpt*) gene. Furthermore, this study innovatively discovered that lonidamine, an inhibitor of cellular energy metabolism, can effectively mitigate SCV resistance to β‐lactam antibiotics, thereby facilitating its eradication. The underlying mechanism involves the reprogramming of purine metabolism. Therefore, a co‐delivery system for lonidamine and oxacillin is constructed with amino‐modified dendritic mesoporous silica as a carrier, which showed high efficacy and safety in combating SCV both in vitro and in vivo experiments. Overall, this study elucidated the pathogenic mechanisms of a class of clinically isolated SCV isolates with *hpt* mutations and provided a paradigm for treating SCV‐associated osteomyelitis by reprogramming purine metabolism.

## Graphical abstract

This study successfully isolated small colony variant (SCV) strains from osteomyelitis samples and found that alterations in purine metabolism, and consequent increases in antibiotic resistance are typical features of SCV. Based on the finding that regulating purine metabolism can enhance SCV sensitivity to β‐lactams, a co‐delivery system of lonidamine and oxacillin is developed to resensitize and further combat SCV.

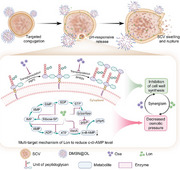

## 1 Introduction

The widespread dissemination of multidrug‐resistant strains has made the clinical treatment of osteomyelitis increasingly challenging.^[^ ^1^ ^]^ Among the most notorious pathogens of osteomyelitis infections is *Staphylococcus aureus* (*S. aureus*), which is capable of making adaptive changes according to external cues.^[^ ^2^ ^]^ In recent years, an adaptive variant of *S. aureus*, small colony variant (SCV), has gained much attention, with evidences linking its presence to the persistence of osteomyelitis.^[^ ^3^ ^]^ SCV is a subset of slow‐growing bacteria distinguished by including smaller colonies, heightened resistance, and diminished hemolytic activity.^[^ ^4^ ^]^ SCV typically evolves from its homologous “normal colony” (NC) strain, potentially mediated by unfavorable factors, including prolonged antibiotic pressure and oxidative stress. However, this mutation is generally unstable. SCV can rapidly revert to the original phenotype either by reversing mutation or acquiring a new mutation at a different site when the survival environment changes. This feature poses a significant challenge in unraveling the mystery of SCV.^[^ ^5^ ^]^ Currently, most of the knowledge about SCV was obtained through studies on SCV‐like model strains, which are constructed by site‐directed mutagenesis.^[^ ^6^ ^]^ Notably, clinically isolated SCV isolates often exhibit significantly different and more complex traits and pathogenic mechanisms.^[^ ^7^ ^]^ This fact implies that research based on clinically isolated stable SCV can provide more insightful information and evidence.

Colonies derived from single‐cell inocula of methicillin‐resistant *S. aureus* (MRSA) have ever been observed to exhibit significant heterogeneity in antibiotic susceptibility, with most colonies demonstrating low to moderate levels of resistance, while a small fraction displays an exceptionally high degree of resistance. This phenomenon was first recognized in 1960 during the microbiological examination of the first documented MRSA infection.^[^ ^8^ ^]^ Actually, SCV, a much more resistant strain than its homologous NC, is one of the most important manifestations of heterogeneity.^[^ ^4^ ^]^ As the largest class of antibiotics, β‐lactam antibiotics were once the first‐line treatment for *S. aureus* infection but have become ineffective with the emergence of SCV.^[^ ^5^, ^9^ ^]^ On the other hand, SCV is easily overlooked in routine microbiological diagnosis due to its slow growth, reduced pigmentation, and smaller hemolytic ring,^[^ ^10^ ^]^ leading to a lack of targeted therapeutic interventions and the persistence of SCV infections.^[^ ^11^ ^]^ Therefore, elucidating the resistance mechanisms of SCV and developing safe and effective potentiators of β‐lactams is a feasible and effective strategy.

Recent studies indicate that certain metabolites, such as indole, nitric oxide, and hydrogen sulfide, can improve bacterial survival in antibiotic‐containing environments, suggesting that increased bacterial resistance may be associated with metabolic changes.^[^ ^12^ ^]^ The product of purine metabolism—Cyclic diadenosine monophosphate (c‐di‐AMP)—is a crucial and ubiquitous second messenger in bacteria, which plays a pivotal role in regulating bacterial growth, division, and cell wall homeostasis.^[^ ^13^ ^]^ More importantly, recent studies suggested that c‐di‐AMP may be directly or indirectly involved in bacterial resistance to β‐lactams.^[^ ^14^ ^]^ Meanwhile, it has been validated that the adaptive mechanisms of SCV are closely linked to metabolic changes.^[^ ^15^ ^]^ Therefore, it can be postulated that altering the flow of purine metabolism to increase c‐di‐AMP levels may constitute a mechanism by which SCV resists β‐lactam antibiotics. It can be further hypothesized that reprogramming purine metabolism may be an emerging strategy to resensitize SCV to β‐lactam antibiotics.

**Scheme 1:**
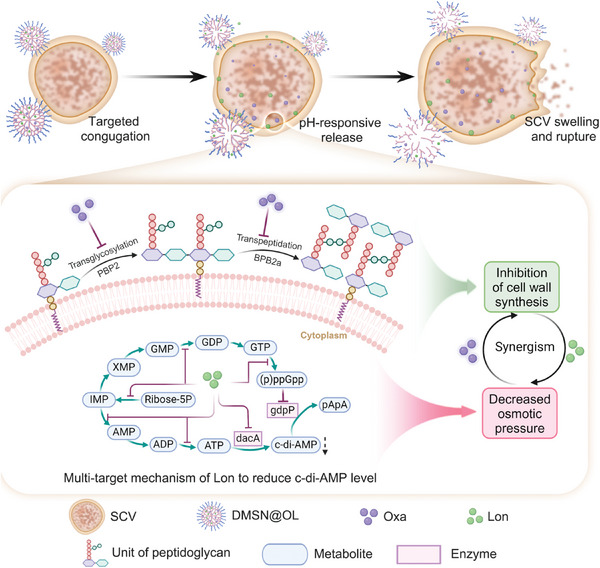
Schematic illustration of the mechanism by which DMSN@OL eradicates SCV. DMSN@OL targets SCV via electrostatic interactions and releases Oxa and Lon in response to a mildly acidic environment. Subsequently, Lon reduces the c‐di‐AMP level through a multi‐target mechanism, leading to decreased osmotic pressure in SCV. This effect works synergistically with Oxa's inhibition of cell wall synthesis. Ultimately, the synergistic action of these two drugs causes the SCV to swell and eventually rupture (Scheme was created with BioRender.com).

Here, five genetically stable SCV isolates have been successfully harvested from patients with chronic osteomyelitis. Mutations in the hypoxanthine phosphoribosyltransferase (*hpt*) gene, one of the key components in the purine salvage pathway, were innovatively found to be a characteristic feature of these strains. Through transcriptomic and metabolomic analyses, we identified key pathogenic mechanisms utilized by these *hpt*‐mutated SCV strains, including enhancing antibiotic resistance by the upregulation of purine metabolism and reducing immunogenicity via the downregulation of virulence factors. Surprisingly, we found that the antiglycolytic agent lonidamine (Lon) intrinsically disrupts purine metabolism in SCV, consequently reducing its resistance to β‐lactams. This effect parallels Lon's established role in modulating energy metabolism within tumor cells. Motivated by the synergistic antibacterial effects of Lon and β‐lactam antibiotics, amino‐modified dendritic mesoporous silica (DMSN) was used as a nano‐delivery carrier for combination therapy, which demonstrated favorable biosafety and efficacy in SCV‐associated osteomyelitis (**Scheme** **1**). Overall, we developed an efficient strategy to alter the flow of purine metabolism in SCV to enhance the efficacy of β‐lactam antibiotics, offering a novel therapeutic approach for the treatment of SCV‐associated osteomyelitis.

## 2 Results and Discussion

### 2.1 *Hpt*‐Mutant Clinically Isolated SCV Strains Featuring High Resistance to β‐Lactams

**Figure 1:**
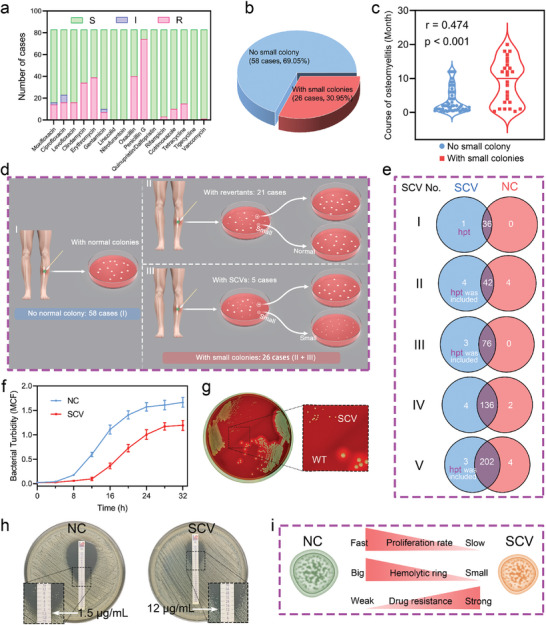
Isolation, identification, and biological characteristics of clinically isolated SCV. a) Antibiotic resistance analysis of clinical *S. aureus* isolates to commonly used antibiotics. S, susceptible; I, intermediate; R, resistant. b) Pie chart showing the quantity and proportion of patients infected with or without small colonies. c) Correlation analysis between small colonies and the course of osteomyelitis by point‐biserial analysis. d) Schematic diagram of the isolation and classification of small colonies of *S. aureus* strains. e) Venn diagram showing the number of genes containing nonsynonymous mutations in genetically stable SCV and the corresponding NC, compared to their respective reference genomes. f) Growth curves of SCV and NC. Results were presented as the mean ± SD, with *n* = 3. g) Image displaying the morphology of SCV and NC on sheep blood agar plate. h) Oxacillin resistance analysis of SCV and NC using E‐test. i) Schematic diagram illustrating the disparities in biological characteristics between NC and SCV.

*S. aureus* strains were isolated from 84 osteomyelitis patients, who were not co‐infected with other bacterial species, at Shanghai Sixth People's Hospital over a period of 9 months. Antibiotic resistance analysis of clinically isolated *S. aureus* strains showed an unfavorable scenario that except for linezolid, nitrofurantoin, and quinupristin/dalfopristin, other types of commonly used antibiotics exhibited varying degrees of resistance (**Figure** **1**a; Data S1, Supporting Information). Remarkably, the resistance to β‐lactams, once considered the gold standard for treating *S. aureus* infections,^[^ ^14a^ ^]^ is particularly concerning. During the identification of bacterial species, we found that a portion (26/84, 30.95%) of *S. aureus* strains were grown accompanied by small colonies upon primary culture (Figure 1b). Notably, the presence of small colonies was significantly positively correlated with the prolonged course of osteomyelitis (Figure 1c). Further analysis of these 26 small colonies showed that most (21/26, 80.77%) reverted to large phenotypes during secondary passage (denoted as revertant). In contrast, a small portion (5/26, 19.23%) maintained their small colony phenotypes in subsequent passages (denoted as SCV) (Figure 1d). These clinically isolated stable SCV strains were essential and employed for subsequent investigations.

Whole‐genome sequencing (WGS) of these five SCV isolates revealed that four carried mutations in the hypoxanthine phosphoribosyltransferase (*hpt*) gene, which occupies a key position in the purine salvage pathway.^[^ ^14a^ ^]^ This indicated that abnormalities in purine metabolism might be a generalized and critical factor driving SCV formation (Figure 1e and Table S1, Supporting Information). To elucidate the mechanism of the *hpt* gene involving SCV formation and its role in pathogenesis, we selected a representative SCV strain harboring solely *hpt* mutation, along with its isogenic NC for further research. The bacterial growth curve showed that SCV had a significantly slower growth rate and ultimately exhibited much lower turbidity in the medium compared to NC (Figure 1f). On sheep blood agar plates, SCV appeared as typical small colonies and lacked the hemolytic rings seen around NC (Figure 1g). In addition, we found a significant eight‐fold difference in minimum inhibitory concentration (MIC) between SCV and NC for Oxacillin (Oxa), a typical β‐lactam antibiotic (Figure 1h). Available investigations have shown that high drug resistance is one of the most prominent features of SCV, which greatly impairs the potency of antibiotics, fueling the refractoriness of osteomyelitis. Figure 1i vividly summarizes the characteristics of SCV in terms of proliferation rate, drug resistance, and hemolytic activity, which are significantly different from those of NC.

### 2.2 SCV Promotes the Persistence of Osteomyelitis by Evading Immune System Attack

**Figure 2:**
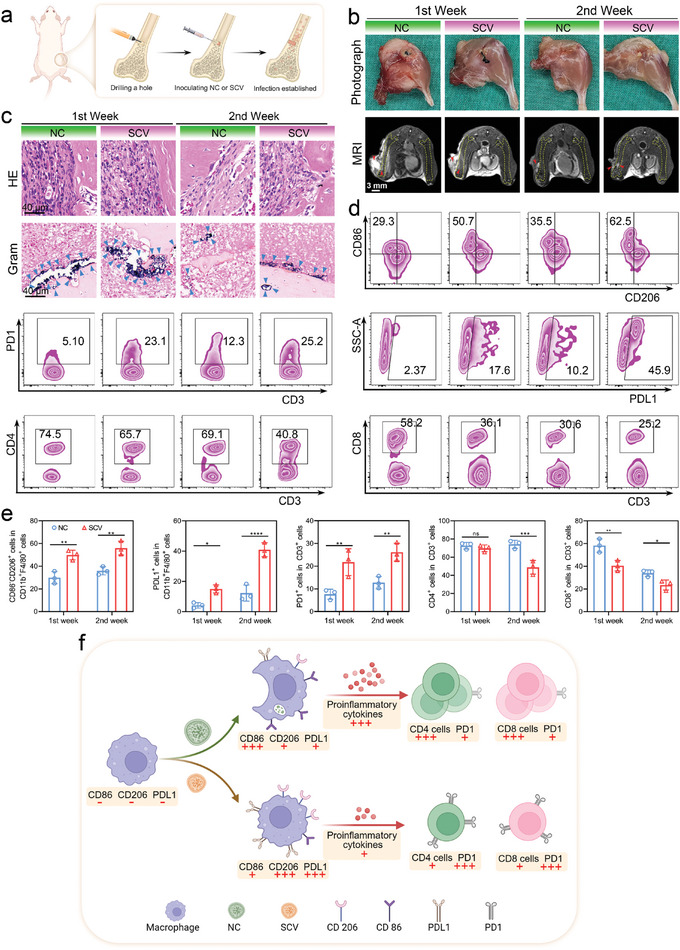
Pathophysiological characteristics of SCV‐related osteomyelitis. a) Schematic diagram depicting the modeling of SCV‐ or NC‐related osteomyelitis. b) Representative photographs and MRI images of infected legs at the end of the first week and the second week. The femur outline is depicted with yellow dashed lines, and the red arrows signify a pronounced inflammatory response c) Representative HE images and Gram staining images of the infected site. The blue triangles indicate the remaining bacteria. d) Representative flow cytometry plots and e) statistical analysis of CD86^−^CD206^+^ cells, PDL1^+^ cells after gating on CD11b^+^F480^+^ cells in the bone marrow, and PD1^+^ cells, CD4^+^ cells, CD8^+^ cells after gating on Gr1^−^CD3^+^ cells in infection‐draining lymph nodes. Results are presented as the mean ± SD, with *n* = 3. f) Schematic illustration depicting the mechanisms that SCV induces a weaker immunological response compared to NC (Schematic illustration was created with BioRender.com).

The host immune response to the pathogens is another crucial factor in determining the outcome of osteomyelitis.^[^ ^16^ ^]^ However, the interaction between SCV and the host immune system remains underexplored. To unravel the mystery, we constructed two osteomyelitis models using NC and SCV following the procedure shown in **Figure** **2**a. After one week, the abscesses on the harvested samples were more pronounced in the NC‐associated osteomyelitis model than in the SCV model, which was further corroborated by a higher T2‐weighted Magnetic Resonance Imaging (MRI) signal. However, as the disease progressed (at the end of the second week), the situation seemed reversed: the abscesses caused by NC were confined to the extramedullary area, while in SCV‐associated osteomyelitis, necrosis, and suppuration were observed both inside and outside the marrow cavity (Figure 2b). Furthermore, in the hematoxylin and eosin (HE) stained section of the SCV group, there was a sustained high level of inflammatory cell infiltration over the 2‐week period, while that of the NC group decreased markedly. These results suggested a relatively mild but persistent infection in the SCV group. Furthermore, we assessed the residual bacteria using Gram staining and standard plate counting test which showed that the quantity of NC was significantly lower than that of SCV, both at the end of the first week and the second week. Additionally, NC exhibited a more pronounced decline than SCV over the 2‐week period (Figure 2c; Figure S1, Supporting information). These results suggested that NC can be cleared by the host in a timely manner, whereas in the SCV‐induced osteomyelitis model, the host's immune response may be compromised or not fully engaged.

The bone marrow harbors a significant population of macrophages, known for their prompt responsiveness and robust phagocytic capabilities, constituting the initial barrier against bacterial invasions.^[^ ^17^ ^]^ Furthermore, macrophages serve as a crucial link in activating adaptive immune cells, which are concentrated in the lymphoid organs.^[^ ^18^ ^]^ To investigate the immunomodulatory properties of SCV, bone marrow from the infected femur and the corresponding draining lymph nodes of mice was extracted for immunophenotyping analysis (Figure 2d,e). Flow cytometry showed that a specific subtype of macrophages (CD86^−^CD206^+^), which hinders bacterial phagocytosis, killing, and the secretion of pro‐inflammatory cytokines,^[^ ^19^ ^]^ remained elevated in cases of SCV‐related osteomyelitis compared to the NC group at the end of both the first week and the second week. This suggested that macrophages are unable to shift toward a bactericidal phenotype in response to SCV promptly. The expression of programmed death ligand 1 (PDL1) on macrophages and programmed death 1 (PD1) on T cells was also higher in the SCV group than in the NC group. The conjugation of PD1 and PDL1 potentially impedes the bactericidal effect mediated by adaptive immunity.^[^ ^20^ ^]^ CD4^+^ T cells play a crucial role in enhancing the phagocytic and bactericidal capabilities of macrophages through the secretion of IFN‐γ, as well as mediating the differentiation of B cells into plasma cells for the production of specific antibodies,^[^ ^21^ ^]^ while CD8^+^ T cells are essential in promptly identifying and eliminating cells that are parasitized by bacteria.^[^ ^22^ ^]^ Our analyses found that the proportion of CD4^+^ T cells in the SCV group decreased after 2 weeks, while CD8^+^ T cells in the SCV group decreased during both time points in comparison with the NC group. This phenomenon indicated that the SCV‐induced immune microenvironment lacks sufficient infiltration of adaptive immune cells with bactericidal capabilities. The ELISA analysis of cytokines in bone marrow fluid demonstrated a reduction in the pro‐inflammatory cytokine TNF‐α and an elevation in the anti‐inflammatory cytokine IL‐10 in the SCV group compared to the NC group (Figure S2, Supporting information). This alteration in the cytokine profile is likely a contributing factor to the persistence of SCV infection.

In summary, the mechanism by which SCV evades the immune system can be preliminarily outlined in Figure 2f. The diminished immunogenicity observed in SCV, potentially stemming from decreased expression of virulence factors, hinders the activation of innate and adaptive immune responses, thereby impeding the prompt eradication of the pathogen. The immune inertness of SCV, combined with its antibiotic resistance, makes the treatment of SCV‐associated osteomyelitis challenging. Therefore, A deeper understanding of the distinct pathogenic mechanisms of SCV may be essential in resolving this medical concern.

### 2.3 Downregulating Immunogenicity While Upregulating Purine Metabolism Constitutes a Critical Pathogenic Mechanism of SCV

**Figure 3:**
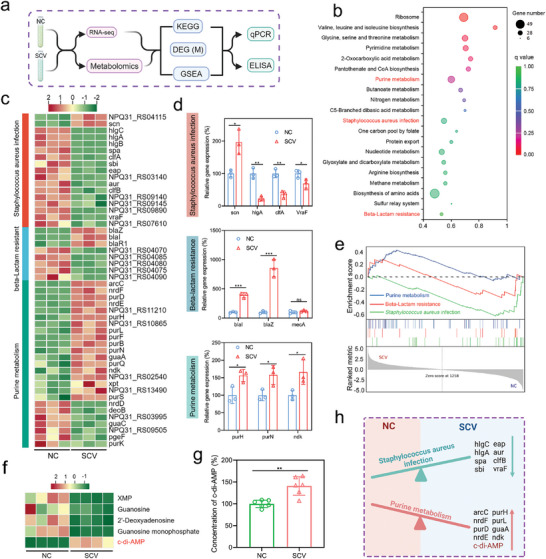
Analysis of pathogenic mechanisms of SCV using RNA‐seq and metabolomics. a) Schematic diagram of the analysis workflow. b) Top 20 KEGG pathways based on DEGs between NC and SCV. c) Heatmap displaying DEGs. d) RT‐PCR validation of selective DEGs in *Staphylococcus aureus* infection pathway, β‐Lactam resistance pathway, and purine metabolism pathway. Results were presented as the mean ± SD, with *n* = 3. e) GSEA enrichment analysis of *Staphylococcus aureus* infection pathway, β‐Lactam resistance pathway, and purine metabolism pathway in RNA‐seq. f) Heatmap of DEMs in purine metabolism. g) Determination of c‐di‐AMP concentration in NC or SCV Using HPLC. Results were presented as the mean ± SD, with *n* = 6. h) Schematic diagram summarizing the transcriptomic and metabolomic differences between NC and SCV. Figure 3a,h are created with BioRender.com.

To elucidate the pathogenic mechanisms of SCV, we conducted transcriptomic and metabolomic analyses, along with related validation tests, as shown in **Figure** **3**a. Principal component analysis indicated that the samples were reproducible within groups and showed variability between groups (Figure S3a, Supporting information). Compared to NC, 501 genes were significantly downregulated and 463 genes were significantly upregulated in SCV. Among them, *hla*, the gene encoding α‐hemolysin, was downregulated, explaining the absence of the hemolytic ring in SCV (Figure S3b, Supporting information). Kyoto Encyclopedia of Gene and Genomes (KEGG) enrichment analysis indicated a significant enrichment of the *Staphylococcus aureus* infection pathway, which is tightly associated with bacterial pathogenesis (Figure 3b). Most genes in this pathway were downregulated, including virulence‐related genes (*hlgC, hlgA, hlgB*) and genes encoding bacterial surface proteins (*spa, clfA, clfB, sbi*). In contrast, a few genes mediating bacterial immune evasion, including *scn*, were upregulated (Figure 3c,d). Additionally, the results of Gene Set Enrichment Analysis (GSEA) indicated a notable downregulation of the *Staphylococcus aureus* infection pathway, with its NES value (−1.92) highlighting a significant role in the SCV phenotypic switch (Figure 3e and Table S2, Supporting Information). These results implied that SCV is inclined to reduce the expression of virulence factors throughout the infection process, which significantly elucidates the phenomenon of immune evasion. On the other hand, although the β‐lactam resistance pathway was included in the top 20 KEGG pathways and contained several significantly regulated genes, it did not exhibit significant regulation in the GSEA analysis (Figure 3e and Table S2, Supporting Information). More importantly, the *mecA* gene, which encodes PBP2a and primarily promotes β‐lactam resistance in *S. aureus*, did not show a significant difference in expression levels between SCV and NC (Figure S3b, Supporting information). Therefore, it can be postulated that the development of resistance in SCV may not be linked to *mecA* and cannot be explained by the modulation of the β‐lactam resistance pathway. Considering the established association between *hpt* and β‐lactam resistance in *S. aureus*,^[^ ^14a^ ^]^ we investigated differentially expressed genes (DEGs) within the purine metabolism pathway. Figure 3d revealed that a substantial proportion of genes in this pathway were upregulated, with a smaller fraction showing downregulation. Concurrently, the GSEA result showed a significant upregulation in purine metabolism (Figure 3e and Table S2, Supporting Information). This suggested that the observed alterations in antibiotic resistance in SCV could potentially be attributed to modifications in the flux of purine metabolism. To verify this hypothesis, the purine metabolism reprogramming was detected by metabolomic analysis. KEGG analysis based on differentially expressed metabolites (DEMs) showed that the purine metabolism pathway was significantly enriched, as indicated by the highest q value (Figure S4, Supporting information). Four metabolites within the purine metabolism pathway displayed a significant upregulation, while c‐di‐AMP exhibited a notable downregulation (Figure 3f). C‐di‐AMP, a pivotal and widespread second messenger in bacteria, plays a crucial role in regulating osmotic pressure, helping to maintain cellular turgor and structural stability.^[^ ^23^ ^]^ Recent research suggested a positive regulatory role of intracellular c‐di‐AMP in bacterial tolerance to β‐lactams.^[^ ^24^ ^]^ Subsequently, we further verified that the c‐di‐AMP level in SCV was significantly higher than in NC by high‐performance liquid chromatography (HPLC) (Figure 3g). As we all know, the primary antimicrobial mechanism of β‐lactams lies in their ability to inhibit bacterial cell wall synthesis, which compromises the bacterial cell's structural integrity, leading to osmotic instability and eventual cell lysis under low osmotic pressure.^[^ ^25^ ^]^ In SCV, the elevated levels of c‐di‐AMP theoretically counterbalance the osmotic stress induced by β‐lactams, reducing the likelihood of cell lysis even when cell wall synthesis is impaired. Therefore, it can be concluded that the resistance of SCV to β‐lactam antibiotics is primarily mediated by c‐di‐AMP rather than *mecA*.

In conclusion, the ability of SCV to evade immune clearance is fundamentally linked to their propensity to downregulate a variety of virulence factors and surface antigens during infection. On the other hand, the increased resistance of SCV to β‐lactam antibiotics can be attributed to the upregulation of purine metabolism, specifically the elevation of c‐di‐AMP levels (Figure 3h).

### 2.4 Lon reduces the resistance of SCV to Oxa

**Figure 4:**
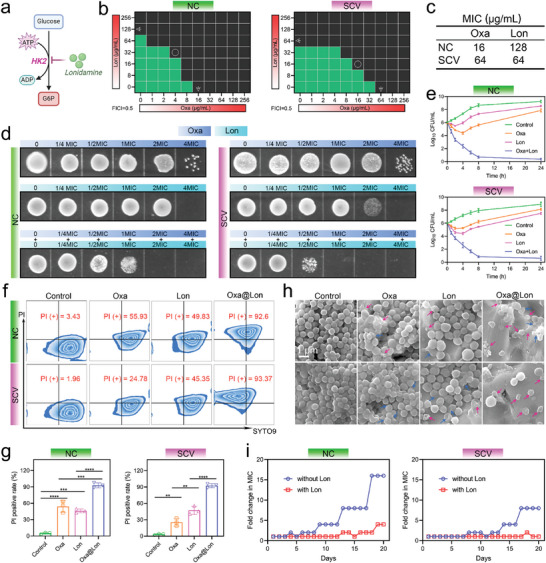
Lon reduces the resistance of SCV and NC to Oxa. a) Schematic diagram showing Lon's effect in disturbing tumor cell metabolism (Schematic diagram was created with BioRender.com). b) Checkerboard broth microdilution assay evaluating the synergistic effect of Lon and Oxa against NC or SCV. The white hollow arrows represent the MIC values of Oxa or Lon, while the white hollow circles indicate the concentrations of Lon and Oxa when bacterial growth is completely inhibited and the FICI value is at its minimum. c) MIC values of Oxa and Lon against NC or SCV. d) MBC test depending on bacterial growth on MH plate after treatment. e) Time‐dependent antimicrobial assay for Lon and Oxa monotherapy or combination therapy against NC or SCV. Results were presented as the mean ± SD, with *n* = 3. f) Representative flow cytometry plots showing PI (+) rate following treatment and g) corresponding statistical analyses. Results were presented as the mean ± SD, with *n* = 3. h) Typical SEM images of NC and SCV after treatment. The pink arrows indicate slight cell deformation and cell membrane shrinkage, while the red arrows indicate cell rupture. i) MIC fold change analysis of Oxa against NC or SCV in the presence or absence of Lon. The concentrations of Oxa and Lon used in treating NC in (e–h) were 32 µg mL^−1^ (2 MIC) and 512 µg mL^−1^ (2 MIC), respectively. For SCV, the concentrations of Oxa and Lon were both 64 µg mL^−1^ (both 1 MIC).

The adaptive survival strategies of SCV render conventional antibiotics ineffective, which presents a significant challenge in the treatment of SCV‐related osteomyelitis.^[^ ^26^ ^]^ To make matters worse, the development of new antibiotics is costly and time‐consuming.^[^ ^27^ ^]^ Considering the pivotal role of purine metabolism in regulating SCV resistance, it is postulated that targeting the flux of purine metabolism to inhibit c‐di‐AMP production may offer a promising approach to overcoming SCV resistance and restoring the efficacy of β‐lactams. Additionally, repurposing non‐antimicrobial agents as antimicrobials or enhancing the effectiveness of existing antibiotics may also be a viable strategy. While screening drugs that can impact SCV purine metabolism, we found inspiration from Lon, an antiglycolytic small molecule known for its ability to disrupt tumor cell metabolism (**Figure** **4**a).^[^ ^28^ ^]^ We sought to utilize Lon to enhance the sensitivity of β‐lactams.

The synergistic effect of Lon and Oxa was observed by a checkerboard broth microdilution assay. As depicted in Figure 4b and Figure S5 (Supporting information), the growth of NC was entirely suppressed at concentrations of 4 µg mL^−1^ for Oxa and 32 µg mL^−1^ for Lon, with a fractional inhibitory concentration index (FICI) of 0.5, indicating a synergistic bactericidal effect of Lon and Oxa on NC. Similarly, the proliferation of SCV was completely inhibited by 16 µg mL^−1^ of Oxa in combination with 16 µg mL^−1^ of Lon, with a FICI value of 0.5, indicating a comparable synergistic effect between the two drugs for SCV. The MIC of the two drugs for NC and SCV are presented in Figure 4c. We observed that the MIC values obtained from the E‐test (Figure 1h) and the checkerboard broth microdilution assay showed significant variation, which may be due to differences in experimental methodologies and observation criteria.^[^ ^29^ ^]^ It should be Subsequently, the minimum bactericidal concentration (MBC) test revealed that NC was effectively eradicated by a combination of 2MIC of Oxa (32 µg mL^−1^) and 2MIC of Lon (256 µg mL^−1^), while the MBC for Lon alone was 4MIC (512 µg mL^−1^) and that for Oxa alone exceeded 4MIC (64 µg mL^−1^). In the case of SCV, a combination of as little as 1 MIC of Oxa (64 µg mL^−1^) and 1 MIC of Lon (64 µg mL^−1^) was adequate for clearance, with the MBC for Lon alone reaching up to 4MIC (256 µg mL^−1^) and the MBC for Oxa alone exceeding 4MIC (Figure 4d). Following this, the time‐dependent antimicrobial assays and flow cytometry experiments demonstrated that the combined treatment of Lon and Oxa exhibited enhanced bactericidal activity compared to the individual drugs at equivalent concentrations for both NC and SCV (Figure 4e–g). Furthermore, scanning electron microscopy (SEM) visually depicted the synergistic antibacterial effects of the two drugs (Figure 4h). Compared to the control group, Oxa or Lon alone resulted in a moderately sparser distribution of bacterial cells in both NC and SCV, with treated bacterial membranes appearing roughening (denoted by blue arrows) or rupture (denoted by red arrows). This tendency was further exacerbated upon co‐treatment, resulting in reduced bacterial adhesion and substantial irreversible harm, such as distortion, perforation, and disintegration. In the antibiotic resistance induction experiment, both NC and SCV strains developed resistance to Oxa after sub‐MIC exposure for 20 consecutive passages, with NC showing a more pronounced resistance trend. However, the introduction of Lon effectively inhibited the development of resistance, implying that synergistic therapeutic approaches could serve as an effective strategy for substantially reducing bacterial resistance (Figure 4i).

The study above confirmed that Lon can efficiently resensitize NC and SCV to Oxa. Notably, for the more challenging SCV, Lon was able to inhibit bacterial resistance at a lower concentration. This synergistic effect presents a promising opportunity to overcome SCV resistance to β‐lactams.

### 2.5 Lon Resensitizes SCV to Oxa by Reversing Purine Metabolism Reprogramming

**Figure 5:**
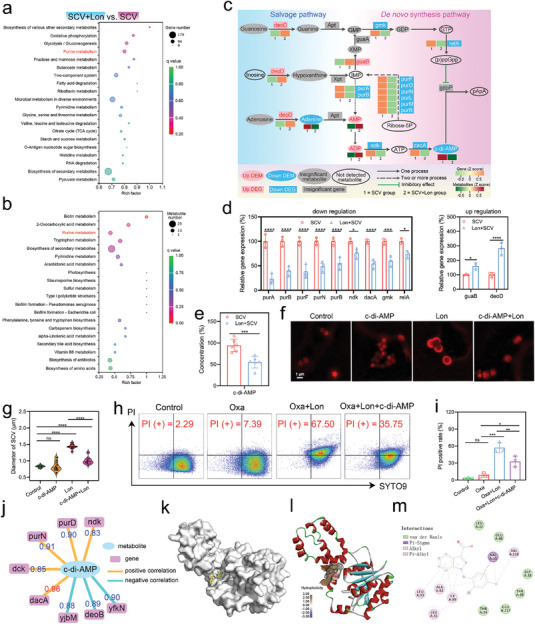
Lon resensitizes SCV to Oxa by reversing purine metabolism reprogramming. Top 20 KEGG pathways based on a) DEGs or b) DEMs between SCV + Lon group and SCV group. c) Purine metabolism pathway showing the involvement of Lon in regulating targeted gene and corresponding metabolites. d) RT‐PCR validation of gene expression in purine metabolism. Results were presented as the mean ± SD, with *n* = 3. e) Determination of c‐di‐AMP concentration using HPLC. Results were presented as the mean ± SD, with *n* = 6 f) Typical microscopic images displaying the sizes of SCV after labeled with Rhodamine‐labeled alanine and g) corresponding statistical analysis. *n* = 20. h) Flow cytometry plots of SCV showing PI (+) rate after treatment and i) corresponding statistical analysis. Results were presented as the mean ± SD, with *n* = 3. j) Correlation analysis of c‐di‐AMP with involved DEGs. The threshold for filtering metabolite‐gene correlations was ∣correlation coefficient∣ > 0.80 and *p*‐value < 0.05. k) Molecular docking analysis for Lon and dacA. l) Binding affinity analysis of the binding site. m) 2D interaction map of dacA and Lon.

With the demonstration that Lon effectively reduces SCV resistance to β‐lactams, further research is necessary to validate the hypothesis that Lon decreases SCV resistance by reprogramming purine metabolism. KEGG analyses of transcriptomics and metabolomics revealed that post‐Lon treatment, the purine metabolism pathway in SCV exhibited significant enrichment among the Top 20 pathways (**Figure** **5**a,b). Furthermore, the NES value (−1.68) of purine metabolism in GSEA analysis indicated its non‐negligible role in SCV phenotype conversion (Figure S6 and Table S3, Supporting Information). In conjunction with prior bioinformatics analysis (Figure 3), we identified shared DEGs and DEMs between the comparisons of SCV versus NC and SCV + Lon versus SCV. K‐means clustering analysis indicated that 14 out of the 21 shared DEGs and the shared DEM, c‐di‐AMP, exhibited a pattern of initial increase followed by a decrease, mirroring the observed changes in bacterial resistance across the three groups (Figure S7 and Tables S4–S6, Supporting Information). This suggested a strong association between Lon‐mediated bacterial resistance and purine metabolism. Additionally, we focused on the β‐lactam resistance pathway in the GSEA analysis, which did not show significant regulation (Figure S8 and Table S3, Supporting Information). RT‐PCR analysis also showed that *mecA* expression remained unchanged before and after Lon treatment (Figure S9, Supporting information). These results implied that the influence of Lon on SCV is likely independent of changes in resistance genes. As illustrated in Figure 5c, lonidamine reduced purine metabolism flux by inhibiting the expression of a series of key genes, including purA, purB, purF, purD, purN, purL, purM, ndk, dacA, and relA, ultimately decreasing the intracellular levels of c‐di‐AMP within SCV. We refer to this as a “multi‐target mode of action”. Figure 5c vividly illustrates Lon's multi‐target regulatory effect on purine metabolism in SCV, which ultimately reduces c‐di‐AMP levels. Subsequently, the validation of this regulatory network by quantitative reverse‐transcription polymerase chain reaction (RT‐PCR) and HPLC also showed consistent results (Figure 5d,e). The findings mentioned above suggested that the effect of Lon in reducing SCV resistance is closely linked to purine metabolism and c‐di‐AMP.

Given that bacterial size is a critical phenotype influenced by c‐di‐AMP,^[^ ^30^ ^]^ the role of Lon was visually validated by staining live SCV cell walls with rhodamine‐labeled alanine. The fluorescent images revealed that Lon markedly increased the average diameter of SCV from 0.87 to 1.43 µm. However, supplementation with c‐di‐AMP effectively mitigated this effect, decreasing the average bacterial diameter to 0.96 µm (Figure 5f,g). In the flow cytometric analysis, the trend in forward scatter area (FSC‐A) also reflected the change in bacterial sizes, which was consistent with the bacterial imaging results (Figure S10, Supporting information). Considering the established view that c‐di‐AMP controls bacterial size, we can conclude that Lon's regulatory effect is mediated by c‐di‐AMP.^[^ ^31^ ^]^ Using live/dead staining, we observed that the reversal of SCV resistance by Lon could be mechanistically counteracted by c‐di‐AMP (Figure 5h,i). It is well recognized that the primary mechanism of action of β‐lactams is to inhibit cell wall synthesis, consequently compromising the bacterial ability to withstand external low osmotic pressure.^[^ ^32^ ^]^ Mechanistically, this process can be further exacerbated by Lon, which regulates c‐di‐AMP to disrupt osmotic pressure homeostasis. This interaction may explain the synergistic antibacterial effect of Lon and oxacillin.

Furthermore, we investigated the specific mechanisms by which Lon regulates c‐di‐AMP in SCV. Correlation analysis revealed a strong association between c‐di‐AMP and the diadenylate cyclase (dacA) gene, indicated by the highest correlation coefficient of 0.96 among the relevant genes (Figure 5j). From a biosynthetic perspective, c‐di‐AMP is synthesized from two ATP molecules by dacA^[^ ^33^ ^]^; therefore, it stands to reason that c‐di‐AMP shows the highest correlation with the dacA. We further used molecular docking and molecular dynamics simulations to investigate whether Lon can directly act on dacA and inhibit its activity. The simulation results showed that Lon binds within a groove at the junction of two subdomains of dacA, exhibiting favorable shape complementarity (Figure 5k). As shown in Figure 5l,m, this binding groove had good hydrophobic properties, and the residues involved included Val218, Val35, Ile89, and Leu93, which together can form strong pi‐alkyl stacking interactions with the benzene ring of Lon to stabilize the binding. The root mean square displacement (RMSD) and radius of gyration (Rg) curve in the kinetics process indicated that the conformation of dacA underwent significant changes during its interaction with Lon and ultimately stabilized after the complex formation (Figures S11 and S12, Supporting information). These results suggested that Lon can tightly bind to dacA to exert its biological effects, which is an important mechanism by which Lon regulates c‐di‐AMP.

In summary, it can be inferred that Lon can modulate c‐di‐AMP levels through multiple targets within bacterial purine metabolism, as depicted in Figure 5f. This multi‐target mode of action allows Lon to effectively mitigate the intrinsic resistance of SCV. Of particular significance among these targets is dacA, whose catalytic function can be disrupted by direct binding with Lon.

### 2.6 Construction of Nano‐Delivery System for Targeted Delivery and Controlled Drug Release

**Figure 6:**
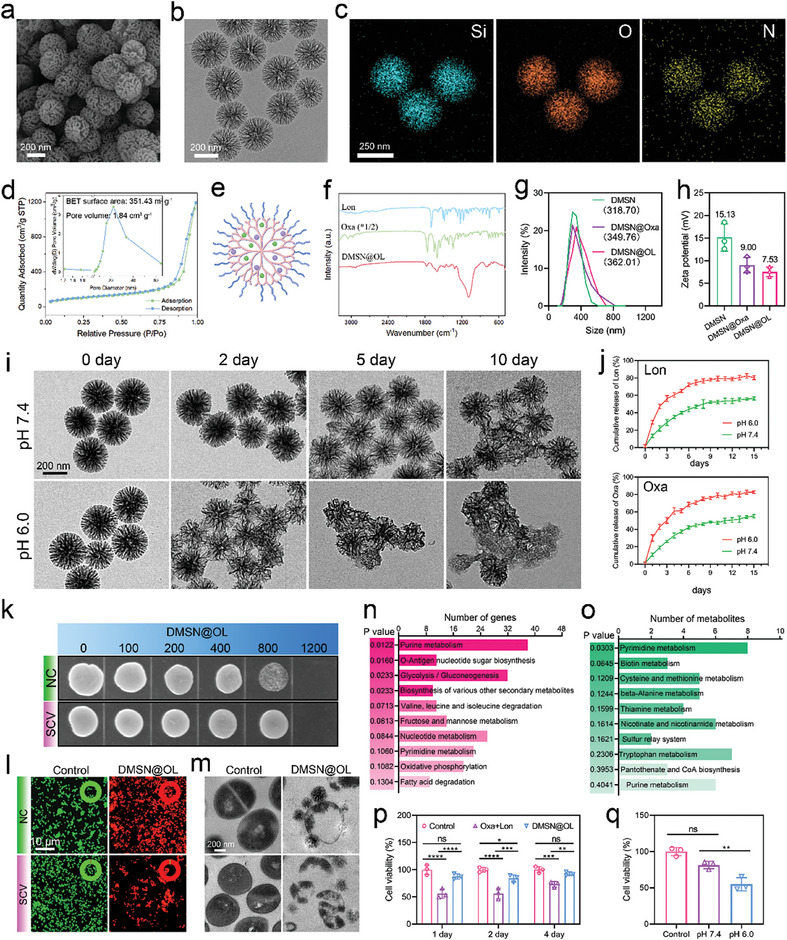
Structural characterization and antibacterial performance evaluation of DMSN@OL. a) Typical SEM image, b) TEM image, and c) elemental mapping images of DMSN. d) N_2_ adsorption‐desorption isotherms of DMSN. e) Schematic diagram of DMSN@OL. f) Fourier infrared spectroscopy of Lon, Oxa, and DMSN@OL. g) Particle size distribution and h) zeta potential of DMSN, DMSN@Oxa, and DMSN@OL. i) TEM images showing the degradation characteristics of DMSN@OL at different pH. j) Drug release profiles of DMSN@OL at different pH. k) MBC test of DMSN@OL based on bacterial growth on MH plate. l) Live/dead staining and m) TEM images of SCV and NC after treated with DMSN@OL. The green and red portions of the circle rings in live/dead staining represent the proportions of live and dead bacteria, respectively. n) Top 10 KEGG pathways enriched in SCV after treated with DMSN@OL based on n) DEGs and o) DEMs. Cell viability of MC3T3‐E1 cells assessed using the CCK‐8 assay after treatment with p) DMSN@OL on days 1, 2, and 4, or with q) DMSN@OL extracts at different pH levels. The results in Figure 6h,j,p,q are presented as the mean ± SD, with *n* = 3.

Osteomyelitis is a challenging infection, with intravenous antibiotic administration often failing to achieve bactericidal concentrations at the infection site.^[^ ^34^ ^]^ Polymethylmethacrylate bone cement has commonly been utilized as a drug release system in clinical practice, yet it is associated with limitations including unstable antibiotic release, incompatibility with heat‐labile antibiotics, and non‐biodegradability.^[^ ^35^ ^]^ Mesoporous silica is emerging as a promising drug delivery system due to its favorable biocompatibility, responsive degradation, ease of surface functionalization, and large pore size for drug loading.^[^ ^36^ ^]^ Considering the specificity of bacterial electronegativity, we modified the surface of these nanoparticles by grafting amino groups, resulting in a positively charged nanocarrier (dendritic mesoporous silica nanoparticles, DMSN). SEM and transmission electron microscopy (TEM) results showed that DMSN was uniformly spherical, monodispersed, with a diameter of ≈290 nm, and exhibited a dendritic nanostructure (**Figure** **6**a,b). The energy dispersive spectroscopy (EDS) pattern exhibited a uniform distribution of Si, O, and N elements, which demonstrated that the synthesis of the nanoparticles and the amino modification were successful (Figure 6c; Figure S13, Supporting Information). Furthermore, N_2_ adsorption‐desorption isotherms further confirmed the mesoporous nature of these DMSN nanoparticles, featuring an average pore size of 22.4 nm and a Brunauer‐Emmett‐Teller surface area of 351.4 m^2^ g^−1^, which provided a favorable structural basis for drug loading (Figure 6d). Subsequently, Oxa and Lon were loaded into the DMSN (denoted as DMSN@OL) by physical pore adsorption, as shown in Figure 6e, with drug loading capacities of 18.13% for Oxa and 28.23% for Lon, respectively. The characteristic peaks observed in Fourier infrared spectroscopy verified the drug payload (Figure 6f). The particle size distribution based on TEM indicated that the size of DMSN did not change significantly before and after drug loading (Figure S14a,b, Supporting Information). However, Figure 6g showed a marginal upward trend in particle size across DMSN, DMSN@Oxa, and DMSN@OL. This may be due to the carboxyl groups forming hydrogen bonds with water molecules through their oxygen atoms, which potentially increases the hydration layer around the particles and could result in a larger hydrodynamic diameter as measured by dynamic light scattering (DLS).^[^ ^37^ ^]^ Additionally, as shown in Figure 6h, the negative charge from the carboxyl groups of oxacillin and lonidamine partially neutralized the positive charge on DMSN. The N₂ adsorption‐desorption isotherms showed that both the specific surface area and pore volume of DMSN‐OL nearly decreased by half after drug loading, which could be attributed to the drug occupying the internal space of the nanoparticles (Figure S14c, Supporting Information). Then, the degradation performance and drug release profiles of DMSN@OL were assessed under pH 7.4 (simulating physiological conditions) and pH 6.0 (simulating the mildly acidic microenvironment associated with osteomyelitis). As shown in Figure 6i, after 2 days of degradation, the dendritic nanostructure of DMSN@OL exhibited slight blurring at pH 7.4, with preservation of its original shape. In contrast, at pH 6.0, the dendritic structure displayed moderate disorder and intertwining, with the nanoparticle framework beginning to deform and even collapse. On the fifth day, acidic conditions drove continued degradation, leading to unclear boundaries between degraded DMSN@OL particles. In contrast, in the neutral environment, DMSN degraded much more slowly, showing only slight deformation and disorder of the dendrites. On the tenth day, the structural integrity of DMSN in a neutral environment experienced gradual collapse and fusion, whereas, in an acidic environment, certain DMSN@OL structures became entirely imperceptible, supplanted by a layer of uniform degradation products. The drug release curves depicted in Figure 6j demonstrated that both drugs exhibited slow release at various pH levels, with faster release occurring in mildly acidic conditions. This pH‐responsive release property enhances bactericidal efficacy and minimizes adverse effects. Results of the MBC test and live‐dead staining indicated that a concentration of 1200 µg mL^−1^ of DMSN@OL was sufficient to effectively eliminate both NC and SCV (Figure 6k,l). TEM showed that DMSN@OL exhibited selective adsorption toward negatively charged bacteria, which facilitated the spatially selective release of antimicrobial drugs, significantly enhancing drug efficiency and reducing side effects (Figure 6m). Transcriptomic and metabolomic analyses further demonstrated that DMSN@OL disturbed various bacterial metabolic pathways, including purine metabolism, pyrimidine metabolism, and glycolysis/gluconeogenesis, which collectively accelerated SCV death (Figure 6n,o).

Subsequently, an assessment of the in vitro biosafety of DMSN@OL was conducted using CCK‐8 assay (Figure 6p). At a concentration of 1200 µg mL^−1^, used to eliminate both NC and SCV, DMSN@OL did not exhibit significant toxicity in MC3T3‐E1 cells during up to 4 days of in vitro culture. In contrast, co‐administration of Oxa and Lon at equivalent concentrations resulted in partial cell death. Additionally, cell viability was assessed following co‐culture with DMSN@OL extracts obtained under different pH conditions. The results indicated that drug release from DMSN@OL was relatively slow and did not cause significant cytotoxicity under neutral conditions (pH 7.4), whereas drug release in an acidic environment (pH 6.0) was faster, leading to a certain level of cytotoxicity (Figure 6q). As purine metabolism is an evolutionarily conserved pathway,^[^ ^38^ ^]^ we assessed the effect of Lon on cellular purine metabolism by measuring ATP levels in osteoblasts, given ATP's essential role as both a precursor and energy source in this pathway.^[^ ^39^ ^]^ The results showed that Lon inhibited ATP production to some extent, thereby interfering with purine metabolism. However, our DMSN‐based drug delivery system significantly mitigated this disruption by enabling a controlled release of Lon (Figure S15a, Supporting information). Importantly, the disturbance in purine metabolism did not affect the expression of genes (ALP, RUNX2, OCN) related to osteogenesis differentiation, which was crucial for bone repair following bacterial clearance (Figure S15b, Supporting information). These results demonstrated the great potential of nano‐delivery systems in mitigating the adverse effects of medications.

### 2.7 Nano‐Delivery Systems can Safely Eliminate SCV and NC and Avoid Infective Osteolysis In Vivo

**Figure 7:**
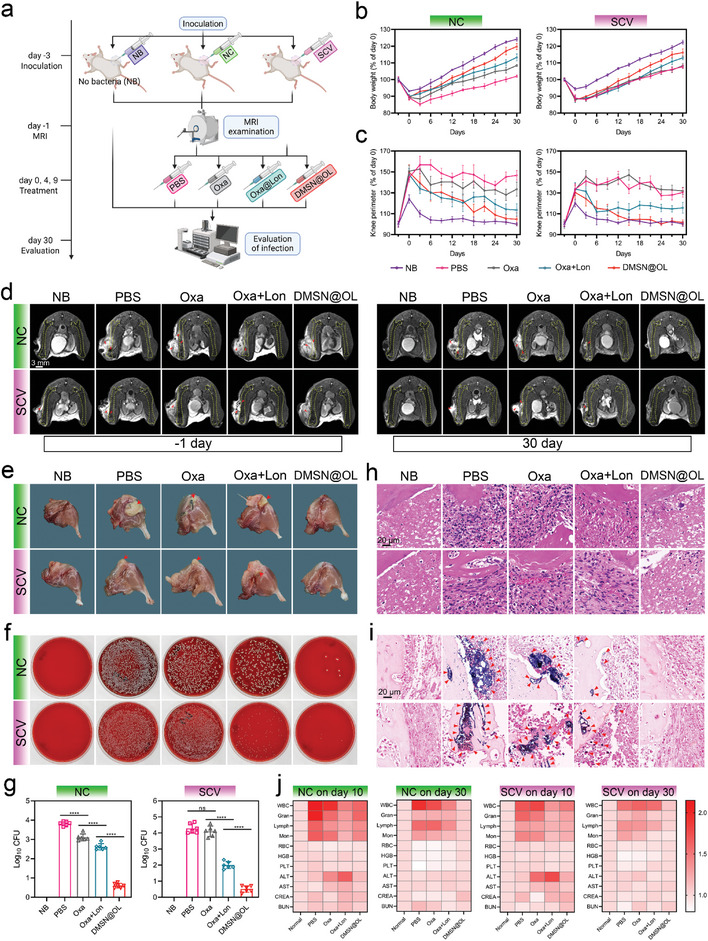
Evaluation of the efficacy and safety of DMSN@OL in the treatment of osteomyelitis. a) Schematic diagram of experimental grouping and treatment regimen (Schematic diagram was created with BioRender.com). b) Body weight changes and c) knee joint circumference changes in NC‐ and SCV‐related osteomyelitis model. Results were presented as the mean ± SD, with *n* = 6. d) Representative MRI images of infected legs before and after treatment. The femur outline is depicted with yellow dashed lines, and the red arrow signifies a pronounced inflammatory response. e) Representative photographs of infected legs after treatment. The red arrows indicate abscesses or tissue necrosis. f) Remaining bacterial counts determined by standard plate counting method and g) corresponding quantitative analyses. Results were presented as the mean ± SD, with *n* = 6. Representative h) HE images and i) Gram staining images of the infected legs. The red triangles indicate the remaining bacteria. j) Assessment of hematological indices on day 10 and day 30.

The efficacy and safety of DMSN@OL in treating osteomyelitis were systematically evaluated by constructing NC‐ and SCV‐infected mouse models. The experiment grouping and treatment regimen are displayed in **Figure** **7**a. The severity of infection during the in vivo experiment was initially assessed by monitoring the body weight of mice, which displayed similar trends in both NC‐ and SCV‐infected models (Figure 7b). From day −3 to day 0, all groups exhibited significant weight loss due to bacterial infection, with the exception of the no bacteria (NB) group, which did not receive bacterial inoculation and only experienced minor weight loss from surgical trauma. Several days post‐treatment, the weight of all groups exhibited an upward trajectory. The DMSN@OL group demonstrated the most rapid weight gain, nearing that of the NB group, while the Oxa+Lon group experienced a comparatively slower increase. Nevertheless, they all surpassed the weight gain observed in the Oxa group and the control group. Concurrently, the circumference of the knee joint was measured to evaluate localized swelling and infection severity, which mirrored the weight findings (Figure 7c). On day −1, the T2‐weighted MRI images showed high signal intensities in the proximal femurs in all groups, indicative of extensive edema and abscess formation, confirming the validation of osteomyelitis. After treatment with a combination of Lon and Oxa (Oxa+Lon group and DMSN@OL group), a more notable decrease in infection signals was observed compared to the other infected groups. Specifically, the DMSN@OL group exhibited superior results in comparison with the Oxa+Lon group in both NC‐ and SCV‐infected models (Figure 7d). Following the euthanasia of the mice, the infected legs were harvested, and the residual bacterial load in infection sites was quantified utilizing the standard plate counting method (Figure 7e–g). In the NC‐related model, a progressive reduction in residual bacterial quantities was observed across the PBS group, Oxa group, Oxa+Lon group, and DMSN@OL group. In the SCV‐related model, the situation was slightly different; there was no significant difference in bacterial content between the PBS group and the Oxa group, suggesting the limited efficacy of Oxa against SCV when used alone. HE staining analysis in both animal models revealed that inflammatory infiltration at infection sites in the DMSN@OL group closely resembled that of the condition without bacterial infection (NB group), suggesting a notable resolution of inflammation compared to the other groups (Figure 7h). In Gram staining, bacteria were stained as blue dots, and their density and distribution also supported the findings above (Figure 7i). These findings indicated that the synergistic therapy combining Oxa and Lon exhibited superior efficacy in treating NC‐and SCV‐associated osteomyelitis compared to Oxa monotherapy. This enhanced therapeutic effect was particularly notable when the agents were co‐delivered using DMSN, which facilitates targeted delivery and responsive release.

In addition, in vivo, biosafety was another crucial consideration for the potential clinical application of nanomedicines. We analyzed the hematological indices of each group at the early stage (day 10) and after the treatment (day 30) (Figure 7j). On day 10, the levels of white blood cell (WBC), granulocyte (Gran), lymphocyte (Lymph), and monocyte (Mon) in the Oxa+Lon group and DMSN@OL group were lower than those in the PBS group and the Oxa group. On day 30, these indices in the DMSN@OL group had decreased to normal levels in both models and were lower than those observed in the Oxa+Lon group. On the other hand, the alanine aminotransferase (ALT) levels in the Oxa group, and especially in the Oxa+Lon group, were elevated on day 10, suggesting a degree of hepatic dysfunction. On day 30, the mice's heart, liver, spleen, lungs, and kidneys were collected and subjected to HE staining, which showed no significant impairments in all the groups (Figure S16, Supporting information). These results suggested that although administering a large dose of drugs may temporarily control the bacterial load, it cannot completely eliminate pathogens under the complex milieu of osteomyelitis and may cause biotoxicity. In contrast, nanocarrier‐mediated targeted delivery and responsive release can effectively reduce these side effects.

**Figure 8:**
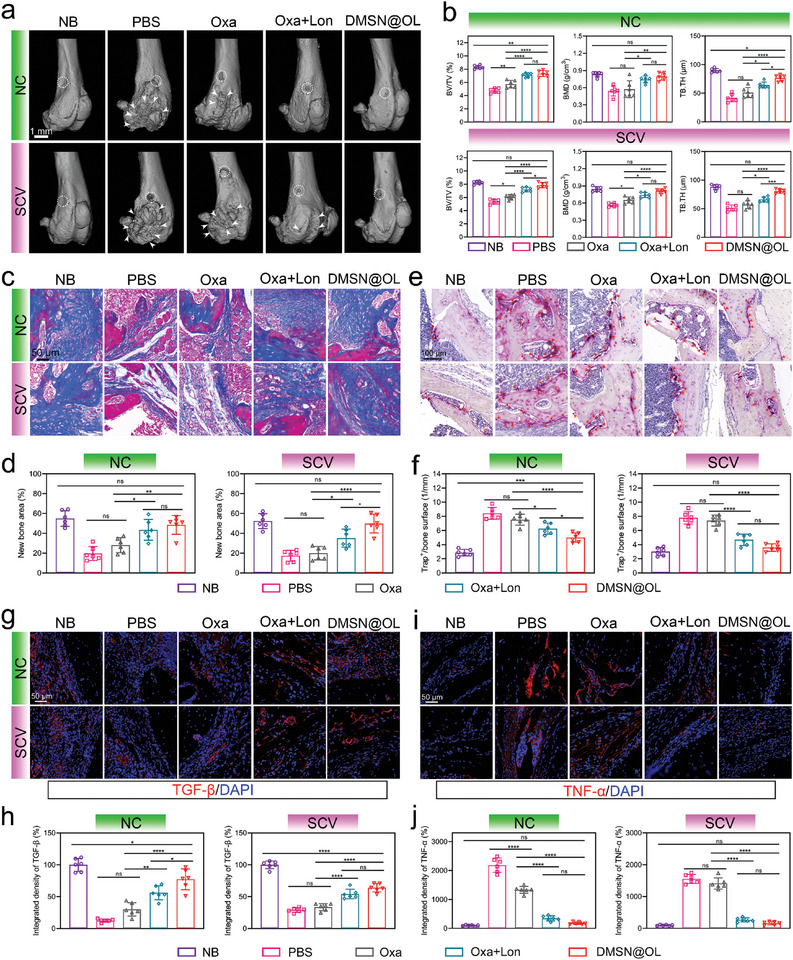
DMSN@OL reshapes the osteogenic microenvironment by timely pathogen clearance. a) Representative micro‐CT images of infected femurs after treatment. The white dashed circles indicate the drilled holes and the white arrows indicate osteolysis. b) Quantitative analyses of micro‐CT parameters, including BV/TV, BMD, and TB.TH. c) Masson staining images of the infected site and d) quantitative analyses of new bone area. e) TRAP staining images of the infected site and f) quantitative analyses of TRAP^+^ cells. The red arrows represent osteoclasts. g) TGF‐β fluorescence staining and h) quantitative analyses of fluorescence intensity. i) TNF‐α fluorescence staining and j) quantitative analyses of fluorescence intensity. The results in Figure 8b,d,f,h,j are presented as the mean ± SD, with *n* = 6.

Infective bone resorption and regeneration disorder is a significant sign of osteomyelitis, posing challenges to the restoration of bone structure and subsequent functional reconstruction.^[^ ^40^ ^]^ Therefore, micro‐CT imaging was employed to evaluate the status of bone resorption and regeneration at the infected site (**Figure** **8**a). Severe bone resorption was observed at the distal femur in both the PBS and Oxa groups, resulting in joint surface collapse and impaired healing of drill holes. Conversely, the Oxa+Lon and DMSN@OL groups exhibited minimal structural damage, with significant healing observed in the latter group. Additionally, the DMSN@OL group demonstrated successful restoration of BV/TV, BMD, and TB. The values (Figure 8b), closely resemble those of the NB group in both NC‐ and SCV‐related osteomyelitis. Masson's trichrome staining was utilized to evaluate the extent of new bone formation, indicated by the blue area. The analysis revealed that the percentage of new bone area was notably higher in both the Oxa+Lon and DMSN@OL groups compared to the other infection groups in the two models. Notably, In SCV‐related osteomyelitis, the osteogenesis in the DMSN@OL group was better than in the Oxa+Lon group (Figure 8c,d). This phenomenon can be attributed to the effective and timely eradication of SCV through the nano‐delivery synergistic approach, which significantly suppressed infection‐induced osteoclast formation and fostered a favorable environment for osteogenesis (Figure 8e,f). To characterize the osteogenic cues in the infection microenvironment, the expression of growth factor TGF‐β and inflammatory factor TNF‐α was examined by immunofluorescence staining (Figure 8g–j). The findings indicated that the DMSN@OL group exhibited a pronounced elevation in TGF‐β levels and a concomitant reduction in TNF‐α levels, suggesting the establishment of a favorable microenvironment conducive to bone regeneration following bacterial clearance.

Given the distinctive physiological characteristics of SCV, including high resistance and low immunogenicity, designing effective strategies to target SCV in the treatment of osteomyelitis often presents a significant and highly formidable issue. In this study, we developed a nano‐delivery system leveraging the synergistic effects of Oxa and Lon, which exhibited improved bactericidal efficacy and safety while inhibiting infective osteolysis in the management of SCV‐related osteomyelitis.

## 3 Conclusion

In conclusion, the heterogeneity of β‐lactam resistance of SCV in osteomyelitis has become a significant concern, yet the underlying mechanisms and therapeutic strategies remain largely unexplored. To the best of our knowledge, this is the first study to reveal that clinically isolated *hpt*‐mutant SCV worsens the prognosis of osteomyelitis by orchestrating increased purine metabolism and decreased immunogenicity. Inspired by the plasticity of purine metabolism‐mediated resistance, we have successfully developed a simple and universal strategy to combat SCV by reprogramming purine metabolism to sensitize β‐lactam antibiotics. This distinctive discovery and universal strategy may initiate a trend of repurposing existing drugs capable of modulating bacterial purine metabolism, thereby mitigating bacterial resistance and influencing the landscape of antibiotic development. Furthermore, this study has demonstrated that the integration of this innovative therapeutic strategy with nano‐delivery systems can effectively address the multifaceted environment of osteomyelitis, thereby enhancing drug efficacy and safety, and exhibiting significant potential for clinical translation.

## 4 Experimental Section

### Ethical Approval

*Staphylococcus aureus* isolates were obtained from patients with informed consent, following approval from the Ethics Committee of Shanghai Sixth People's Hospital (Approval No. 2023‐KY‐017(K)‐(1)). All animal experiments were carried out in accordance with the guidelines and regulations set forth by the Institutional Animal Care and Use Committee (IACUC) of Shanghai Sixth People's Hospital (Animal Welfare Ethics acceptance number no. DWLL2024‐0883).

### Statistical Analysis

All statistical metrics included data from at least three independent samples, presented as mean ± standard deviation, with statistical differences analyzed using Origin 2022 (OriginLab, MA, USA) and GraphPad Prism v9.0.0 (La Jolla, CA, USA). The primary statistical methods used were one‐way ANOVA and Student's *t*‐test. The correlation between infection course and small colonies was calculated by point‐biserial correlation analysis. Statistical significance was denoted as * (*p* < 0.05), ** (*p* < 0.01), *** (*p* < 0.001), and **** (*p* < 0.0001).

## Conflict of Interest

The authors declare no conflict of interest.

## Author Contributions

T.S., Q.W., and Z.R. contributed equally to this manuscript. T.S., M.G., and Y.C. designed this work. T.S. and Q.W. collected clinical *S. aureus* strains. T.S., Z.R., and M.G. performed the experiments. Z.L., W.W., and Z.G. assisted with the animal experiments. Y.M., X.W., and G.C. conducted the data analysis. T.S. wrote the initial manuscript draft. H.L., Y.C., and M.G. supervised the project, revised the manuscript, and commented on it.

## Supporting information



Supporting Information

Supporting Information

## Data Availability

The data that support the findings of this study are available in the supplementary material of this article.
