# Supplementary material for: Resensitizing β‐Lactams by Reprogramming Purine Metabolism in Small Colony Variant for Osteomyelitis Treatment
Source: Adv Sci (Weinh). 2024 Dec 10;12(5):2410781. doi: 10.1002/advs.202410781 (PMC11791937; doi:10.1002/advs.202410781)
Supplement: Supplementary file 1 — Supporting Information [file ADVS-12-2410781-s002.docx]

Supporting Information

**Resensitizing β-lactams by Reprogramming Purine Metabolism in Small Colony Variant for Osteomyelitis Treatment**

Tingwang Shi^1§^, Qiong Wu^4§^, Zesong Ruan^1§^, Zhiyuan Luo^1^, Wenbo Wang^1^, Zhao Guo^1^, Yihong Ma^1^, Xin Wang^1^, Guangyu Chu^5,6^, Han Lin^3^, Min Ge^2^*, Yunfeng Chen^1^*

1. Department of Orthopedic Surgery, Shanghai Institute of Microsurgery on Extremities, Shanghai Sixth People’s Hospital Affiliated to Shanghai Jiao Tong University School of Medicine, 600 Yishan Road, Shanghai, 200233, China

2. Department of Electrical and Electronic Engineering, The University of Hong Kong, Pokfulam Road, Hong Kong, China.

3. Shanghai Institute of Ceramics Chinese Academy of Sciences; Research Unit of Nanocatalytic Medicine in Specific Therapy for Serious Disease, Chinese Academy of Medical Sciences, Shanghai, 200050, China.

4. Department of Laboratory Medicine, Shanghai Sixth People’s Hospital Affiliated to Shanghai Jiao Tong University School of Medicine, 600 Yishan Road, Shanghai, 200233, China

5. Spine Lab, Department of Orthopedic Surgery, The First Affiliated Hospital, Zhejiang University School of Medicine, Hangzhou, 310003, China.

6. Joslin-Beth Israel Deaconess Foot Center and The Rongxiang Xu, MD, Center for Regenerative Therapeutics, Beth Israel Deaconess Medical Center, Harvard Medical School, Boston, MA 02215, USA.

§ Tingwang Shi, Qiong Wu and Zesong Ruan contributed equally to this manuscript.

*Corresponding Author. Min Ge: gmin@mail.ustc.edu.cn; Yunfeng Chen: chenyf@sjtu.edu.cn

**The PDF file includes:**

Methods

Supplementary Figure S1-S18

Tables S1 to S8

Reference 1-6

**Methods**

***Isolation of SCV.*** Patients with osteomyelitis caused by *Staphylococcus aureus*, without co-infection by other bacterial species, were included in this study conducted at Shanghai Sixth People’s Hospital. Bacterial samples from patients were uniformly distributed on 5% sheep blood agar plates and incubated at 37°C for 24 hours. Species identification and minimum inhibitory concentration (MIC) determination were performed using the VITEK 2 automated system (BioMérieux, Marcy l'Étoile, France) according to the manufacturer’s instructions. In cases where both normal and small colonies were observed on blood agar plates during the primary culture, a minimum of three small colonies were randomly selected and subcultured on new plates. Small colonies that reverted to a normal phenotype after the second passage were classified as revertant, whereas those that retained their small morphology were designated as small colony variant (SCV). Additionally, point-biserial correlation analysis was employed to examine the relationship between the duration of infection and the presence of small colonies in the primary culture.

***Whole-genome sequencing (WGS).*** Total genomic DNA from SCV and its corresponding NC was extracted using the TIANamp Bacterial DNA Kit (Tiangen, Beijing, China), followed by agarose gel electrophoresis to confirm DNA quality. Sequencing libraries were then constructed and sequenced using the Illumina NovaSeq 6000 at Hangzhou Kaitai Biotechnology Co., Ltd. (Hangzhou, China). The Mash tool^[1]^ was used to identify the most closely matched reference genome. Data analysis was conducted on a free online platform (https://kaitai.cloud/tools).

***Growth curve.*** The No. I SCV and its corresponding NC were inoculated in TSB and incubated overnight at 37°C. The cultures were then diluted 1,000-fold and further cultivated in a shake flask at 37°C. Bacterial turbidity was measured every 4 hours using a WGZ-XT turbidity meter (Qiwei, Hangzhou, China).

***E-test.*** NC and SCV were inoculated into Mueller-Hinton (MH) medium. After incubation overnight, the turbidity of the bacterial suspension was adjusted to 0.5 McFarland standard (equivalent to 1.5 × 10^8^ cfu/mL). A sterile swab was used to obtain a small amount of bacterial suspension, which was then evenly spread on MH agar plate. After the surface of the medium had dried, an oxacillin (Oxa) E-test strip (Liofilchem, Italy) was placed on the agar with the scale facing up. The agar plate was then incubated upside down for 24 hours. Finally, the MIC was determined based on the position of the inhibition zone.

***Construction of mouse osteomyelitis.*** The BALB/c mice used in this study were bred and housed in the Animal Experimental Center of Shanghai Sixth People’s Hospital. Twenty-four male mice, aged 6-8 weeks, were randomly assigned to two groups to develop NC-related and SCV-related osteomyelitis models. The modeling procedure was conducted as follows: the mice were anesthetized using inhalation anesthesia with 3% isoflurane for induction and 1.5% isoflurane for maintenance. The right leg of each mouse was shaved and subsequently disinfected. A longitudinal incision was made on the anterolateral aspect of the distal femur, followed by meticulous layer-by-layer dissection of the muscle and fascia until the bone surface was exposed. A unicortical hole was created using a mini electric drill equipped with a 27G needle. Through this aperture, 10 µL of 1 × 10^6^ CFU/mL suspension of either NC or SCV was administered into the bone marrow cavity via a microinjector. The bone defect was subsequently sealed with bone wax, and the muscle and skin were sutured in a layered fashion. Finally, the skin surface was disinfected again.

***Evaluation of bone infection.*** One and two weeks after surgery, bone infections in mice were evaluated utilizing a small animal MRI scanner (CG NOVILA 7.0T, Shanghai Chenguang Medical Technologies Co., Ltd) with T2-weighted imaging sequences. At these designated time points, six mice per group were euthanized, and the infection status was documented by taking photographs. Three of these mice were allocated for the preparation of histological sections, while the remaining three specimens were utilized for bone marrow extraction, which was then analyzed using flow cytometry, enzyme-linked immunosorbent assay (ELISA) and standard plate counting.

For histological analysis, samples were initially fixed in 4% formaldehyde and subsequently decalcified in an EDTA solution (Servicebio, Wuhan, China) for two weeks. Following decalcification, the samples underwent dehydration, paraffin embedding, and sectioning into 5-µm thick slices for further staining and microscopic evaluation. The sections were stained with hematoxylin and eosin (H&E) to assess inflammation and with Gram stain to detect remaining bacteria.

For standard plate counting, muscles attached to the femur were carefully removed, both ends of the femur were cut, and the marrow cavities were flushed three times with 5 mL of HBSS buffer (containing 5 mM EDTA and 1% FBS). One milliliter of harvested flush solution was used for standard plate counting. After serial dilution, 100 microliters of each sample at various concentration gradients were evenly spread on 5% sheep blood agar plates, which were then incubated inverted at 37℃ for 24 hours. Lastly, the colonies were counted and analyzed statistically.

***Immunological assessment.*** For the ELISA test, the remaining 4 mL of bone marrow flush solution was carefully filtered through a 70 µm cell strainer (BD Biosciences, California, USA) and then centrifuged. The supernatants were collected for TNF-α and IL-10 content determination according to the ELISA kit instructions (Anogen, Mississauga, Canada). For flow cytometry analysis, erythrocytes in the cell pellet were removed using a red blood cell lysis solution (BD Biosciences). The resulting single-cell suspension was incubated with anti-CD16/32 on ice for 20 minutes to block Fc receptors. After washing, the single-cell suspension was treated with Fixable Viability Dye eF780 and antibodies targeting CD45, CD11b, F4/80, CD86, and PDL1, then incubated on ice for an additional 30 minutes. After washing, the stained cells were fixed and permeabilized utilizing the Cytofix/Cytoperm kit (BD Biosciences) and staining with a CD206 antibody. Subsequently, a final wash was performed to eliminate excess stain, and the single-cell suspension was analyzed via flow cytometry (Beckman Coulter, Brea, CA, USA). Data analysis was conducted using FlowJo software (version 10), with gating strategies illustrated in Figure S17.

The inguinal lymph nodes on the infected side were also collected for analysis. First, the lymph nodes were minced into the smallest possible fragments using a sterile scalpel, followed by digestion with 1 mg/mL collagenase IV (Sigma-Aldrich, USA) and 100 µg/mL deoxyribonuclease (Sigma-Aldrich, USA) at 37℃ for 30 minutes. After obtaining a single-cell suspension through a cell strainer, red blood cells were lysed, and the cells were blocked and washed as described above. The cells were then incubated on ice with Fixable Viability Dye eF780 and antibodies against CD45, CD11b, Gr1, CD3, CD4, PD1, and CD8 for 30 minutes. The detection and analytic methods followed the protocols previously described, and the gating strategy is shown in Figure S12. Additionally, the antibodies used are listed in the Table S7.

***RNA-seq and metabolomics (SCV vs. NC).*** NC and SCV were grown in TSB until reaching the logarithmic growth phase, then the bacterial pellets were collected by centrifugation and quickly frozen in liquid nitrogen. Total RNA extraction, cDNA library construction, and subsequent high-throughput sequencing were conducted by Novogene Bioinformatics Technology Co., Ltd. (Beijing, China). For untargeted metabolomic analysis, liquid chromatography-tandem mass spectrometry (LC-MS/MS) was performed following metabolite extraction by Wuhan Maiteville Biotechnology Co., Ltd. (Wuhan, China). The resulting transcriptomic and metabolomic data were subjected to principal component analysis (PCA), differential expression analysis, and Kyoto Encyclopedia of Genes and Genomes (KEGG) analysis using Metware cloud platform (https://magic.novogene.com/). DEGs were identified based on the criteria of *q* value<0.05 and |log2FC| ≥ 0.5850, while DEMs were identified based on the criteria of *q* value < 0.05, |log2FC| ≥ 0.5850 and VIP ≥ 1. Each group in the RNA-seq analysis contained three biological replicates, while the metabolomics analysis contained four.

***RT-PCR.*** Two milliliters of NC or SCV suspension in the logarithmic growth phase were collected, centrifuged at 3000 rpm for 5 minutes, and washed three times to obtain bacterial pellets. Total bacterial RNA was then extracted using the RNAprep Pure Cell/Bacteria Kit (Tiangen, Beijing, China) following the manufacturer's instructions. Equal amounts of total RNA were reverse transcribed into cDNA using the Color Reverse Transcription Kit (EZBionscience, Roseville, CA, USA). The cDNA from each group, combined with 2× Color SYBR Green qPCR Master Mix (EZBionscience) and primers (Sangon, Shanghai, China), was used for real-time quantitative PCR on the QuantStudio 7 Flex System (Life Technologies, CA, USA). The 2^-ΔΔCt^ method was employed to calculate gene expression levels, with 16S rRNA serving as the reference gene. The primer sequences for the genes are listed in Table S3.

***Determination of c-di-AMP concentration by HPLC.*** The intracellular concentration of c-di-AMP in NC and SCV was quantified using high-performance liquid chromatography (HPLC) with some modifications to established methods^[2]^. Briefly, bacterial pellets were harvested from logarithmic growth phase bacterial suspensions through centrifugation and washing. The extraction process included resuspending bacterial pellets in extraction buffer (acetonitrile, methanol, and water in a 2:2:1 ratio), rapidly freezing in liquid nitrogen for 30 seconds, boiling for 10 minutes, and lysing by bead beating for 45 seconds. The lysate was then centrifuged at 17,000 g for 5 minutes to collect the supernatant. The extract was vacuum freeze-dried and resuspended in sterile water. A 10 µL aliquot of the solution was used to separate c-di-AMP with a reverse-phase HPLC system equipped with a C-18 column (Phenomenex, CA, United States) on a Waters 2695 Separations Module (Waters Corp., Milford, ME, USA). Then c-di-AMP was detected at 254 nm. Quantification was achieved by constructing a standard curve based on a series of standard c-di-AMP solutions with known concentrations. Finally, the c-di-AMP concentration was normalized to the wet weight of the bacteria.

***Checkerboard broth microdilution assay.*** A checkerboard broth microdilution assay was employed to assess the potential synergistic effects of Oxa and lonidamine (Lon), following established protocols^[3]^. In brief, a 10 × 7 matrix was prepared in a V-bottom 96-well plate by combining two-fold serial dilutions of Oxa (ranging from 1 to 256 µg/mL) and Lon (ranging from 8 to 256 µg/mL), as shown in Figure S5. Subsequently, bacterial suspension was introduced into each well to achieve a final concentration of 5 × 10^6^ CFU/mL. Following an 18-hour incubation at 37°C, the MIC was determined based on the presence or absence of visible bacterial colonies. The interaction between Lon and Oxa was assessed by determining the fractional inhibitory concentration index (FICI) using the equation provided below:

$$FICI=\frac{MIC of Oxa in combination}{MIC of Oxa alone}+\frac{MIC of Lon in combination}{MIC of Lon alone}$$

The interaction between the two drugs was determined according to the following criteria^[4]^: FICI ≤ 0.5 indicates synergistic effect, 0.5 < FICI ≤ 4 indicates no interaction, and FICI > 4 indicates antagonistic effect.

***Minimum bactericidal concentration (MBC) test.*** Bacterial growth under varying concentrations of Lon, Oxa, or their combinations was investigated to determine the MBC. Specifically, logarithmic-phase NC or SCV cultures were harvested and adjusted to a turbidity of 0.5 McFarland standard. After adding a series of concentrations of Lon and Oxa according to Figure 4d, the cultures were incubated at 37°C for 24 hours. Subsequently, 10 µL of bacterial suspension from each sample was aspirated and plated onto MH agar plates and incubated for an additional 24 hours. The lowest concentration at which no colonies grew was determined as the MBC for the combination treatment of the two drugs.

***Synergistic bactericidal effects of Lon and Oxa.*** To further investigate the synergistic bactericidal effects of Oxa and Lon, bacterial growth curves were plotted. Briefly, logarithmic-phase NC or SCV cultures were harvested and adjusted to a turbidity of 0.5 McFarland standard. NC cultures were treated with 2MIC of Oxa (32 µg/mL), 2MIC of Lon (256 µg/mL), or a combination of both, while SCV cultures were treated with 1MIC of Oxa (64 µg/mL), 1MIC of Lon (64 µg/mL), or a combination of both. After incubation for 24 h in a 37°C shaking incubator, bacterial counts were determined every 4 hours using standard plate counting.

For flow cytometry analysis, the bacteria treated as previously described were harvested. Following the protocol outlined in the LIVE/DEAD BacLight Viability Kit (Thermo Fisher Scientific, Waltham, MA, USA), working solutions of SYTO9 and propidium iodide (PI) were prepared and subsequently added to the samples. After a 30-minute incubation, the PI-positive rate in each sample was determined using flow cytometry, and the resulting data were analyzed using FlowJo software.

For scanning electron microscopy (SEM) analysis, NC or SCV were inoculated on the surface of titanium discs. After the aforementioned treatments, the titanium discs were fixed with 2.5% glutaraldehyde, dehydrated with a series of graded concentrations of ethanol, freeze-dried, and sputter-coated with gold. Finally, the samples were observed and imaged using a Field Emission Scanning Electron Microscope (JSM-7800F, JEOL, Japan).

***Resistance induction test.*** The microdilution broth method was used to test the induction of bacterial resistance by Oxa in the presence or absence of Lon over a 20-day culture period. Briefly, bacteria at a concentration of 5 × 10^6^ CFU/mL were co-cultured with two-fold serial dilutions of Oxa (1-256 µg/mL) in the presence or absence of Lon (at 1/4 MIC). After 24 hours of incubation, the MIC of Oxa was determined. Subsequently, bacteria treated with 1/2 MIC Oxa were diluted and used for the next cycle of the experiment following the same method. This process was repeated for 20 consecutive days to observe changes in resistance to Oxa in NC or SCV.

***Mechanism analysis of Lon enhancing the antibacterial activity of Oxa.*** Logarithmic-phase SCV cultures were adjusted to a turbidity of 0.5 McFarland standard and treated with Lon (64 µg/mL) for 24 hours. The bacteria were then collected for subsequent experiments, with untreated SCV serving as the control. Transcriptomics, metabolomics, and data analysis were conducted according to previously described methods. RT-PCR experiments and c-di-AMP concentration measurements were also performed as previously described.

***Molecular docking.*** The structure of Lon was downloaded from the PubChem database (http://pubchem.ncbi.nlm.nih.gov/) and converted to PDB format using PyMOL 2.2.0 (http://www.pymol.org/). Subsequently, AutoDockTools 1.5.6 (http://autodock.scripps.edu) was used to load the PDB files, assign atom types, add atomic charges, and save the files in PDBQT format to serve as the ligands for molecular docking. The 3D structure of dacA (ID: Q2FW92.1.A) was downloaded from the AlphaFold database (https://alphafold.ebi.ac.uk/). This structure was also loaded into the AutoDockTools program and saved in PDBQT format to be used as the receptor in molecular docking. Molecular docking was performed using AutoDock Vina (version 1.1.2). In this process, the ligand was set as flexible, while the receptor was kept rigid. The exhaustiveness parameter was set to 100, while all other parameters were kept at their default settings. The center coordinates of the docking box were specified as follows: x = -2.679, y = 3.38, z = 4.417, with each dimension having a size of 50. The docking conformation with the highest score (affinity = -6.5 kcal/mol) was selected and subjected to a 100 ns molecular dynamics simulation to analyze the stability and interactions of the ligand-receptor complex.

***Molecular dynamics simulations.*** Molecular dynamics simulations of the protein-ligand complexes were performed using Amber18 software^[5]^. The protein dacA was parameterized using the ff14SB force field, while the ligand Lon was parameterized using the GAFF. The AM1-BCC atomic charges for Lon were calculated using the ANTECHAMBER module. The protein-ligand complex was loaded into the TLeap module, where hydrogen atoms and counterions were automatically added to neutralize the system. The TIP3P explicit water model was chosen, and periodic boundary conditions were applied. The simulation workflow included energy minimization, heating, equilibration, and production. Initially, the protein and ligand heavy atoms were constrained, and the system underwent 10,000 steps of energy minimization (5,000 steepest descent followed by 5,000 conjugate gradient). The constraints were then released for another 10,000 steps of energy minimization. Subsequently, the system was gradually heated to 300K over 50 ps, equilibrated for 50 ps under NPT conditions, and subjected to a 100 ns molecular dynamics simulation with a 2 fs timestep. Trajectories were saved every 20 ps and analyzed using the CPPTRAJ. Binding free energy calculations were performed using the MMPBSA.py module.

***Determination of bacterial size and PI-positive rate.*** Logarithmic-phase SCV suspension was diluted to 1 × 10^6^ CFU/mL and treated with 30 μM c-di-AMP (MCE, USA), 32 μg mL^-1^ Lon (MCE, USA), or a combination of both for 24 hours. The bacteria were centrifuged, washed, and incubated in the dark with 500 µM rhodamine-labeled alanine (Xian Ruixi Biological Technology Co., Ltd) for 30 minutes. Subsequently, the bacteria were washed with PBS, resuspended, and a small amount of the suspension was added dropwise onto cell slides for observation using a THUNDER Imager (Leica Microsystems, Germany). For FSC-A analysis, the treated bacterial suspension was directly analyzed using a flow cytometer. For PI-positive rate assessment, bacteria after corresponding treatment were analyzed by flow cytometry after staining with SYTO9 and PI.

***The synthesis and characterization of DMSN@OL.*** The dendritic mesoporous silica nanoparticle (DMSN) carrier was synthesized according to published literature,^[6]^ and its surface was modified with amino groups using (3-aminopropyl) triethoxysilane (APTES). Briefly, 0.068 g of triethanolamine (TEA) was added to 25 ml of water and stirred in an oil bath at 80 °C for 30 min. Next, 380 mg of cetyltrimethylammonium bromide (CTAB) and 168 mg of sodium salicylate were added to the solution, and stirring was continued for 60 min; this mixture was designated as solution A. Then, 4 ml of tetraethyl orthosilicate (TEOS) was added to solution A and stirring was continued for 2 h. The product was collected by high-speed centrifugation (13000 rpm) and washed several times with ethanol to remove residual reactants. The collected product was then extracted 3 times with a hydrochloric acid and methanol solution at 60.°C to remove the template and then dried under vacuum at room temperature overnight. For surface amino modification of DMSN, 3 mL of ethanol was added to 1.5 mL of APTES and stirred evenly, followed by the addition of 1 g of DMSN suspended in 4 mL of ethanol. The whole suspension was stirred at room temperature for 24 hours. Subsequent centrifugal washing yielded amino-modified DMSN. All chemicals were used as received without purification.

SEM photographs were obtained on a field-emission Gemini 300 microscope (ZEISS, Germany). Transmission electron microscope (TEM) and high-resolution transmission electron microscope (HRTEM) photographs were acquired on a JEM-2100F transmission electron microscope (JEOL, Japan). The FTIR analysis was performed on Thermo Scientific Nicolet iS20 (Thermo, US). The structure and the pore size of DMSN and DMSN@OL were characterized by N_2_ adsorption-desorption isotherm on a Micromeritics APSP 2460 system (Micromeritics, US). Dynamic light scattering (DLS) measurements were performed on Zetasizer Nanoseries (Nano ZS90, Malvern Instrument Ltd.). For drug loading, 10 mg Lon and 6 mg Oxa were slowly added to a methanol suspension of DMSN (5 mL, 4 mg/mL) and stirred overnight at room temperature. The obtained DMSN@OL NPs were then collected by centrifugation and washed several times. The ability of DMSN to load drugs was determined by measuring the concentrations of Lon and Oxa in the supernatant with UV-VIS analysis (Shimadzu, Tokyo, Japan).

***Degradation and drug release characteristics of DMSN@OL.*** DMSN@OL was dispersed in PBS and adjusted to a concentration of 1200 μg mL^-1^. Then, 5 mL of the dispersion was adjusted to pH 6.0 and 7.4, respectively, and incubated at 37°C for 24 hours. After centrifugation (8000 rpm, 5min), the supernatant was collected for subsequent analysis. The DMSN@OL was then resuspended in fresh PBS to facilitate continued drug release. This procedure was repeated daily for 15 days. The absorbance of the supernatant at 300 nm and 240 nm was measured using UV-vis spectroscopy. The Lon concentrations of samples were determined by OD_300_ using a standard curve established from a series of graded concentrations of Lon solution, while the Oxa concentrations were determined by OD_240_ (with prior deduction of Lon’s absorbance at 240 nm) using a standard curve, similarly. Additionally, on days 0, 2, 5, and 10, a small amount of solution was added dropwise to an ultrathin copper mesh to observe the morphological changes of DMSN@OL using a JEM-2100F field emission TEM (JEOL, Ltd, Tokyo, Japan).

***MBC determination of DMSN@OL.*** Logarithmic-phase NC or SCV cultures were adjusted to a turbidity of 0.5 McFarland standard and then treated with a serial concentration of DMSN@OL (100, 200, 400, 800, and 1200 μg mL^-1^) for 24 h. Subsequently, 10 μL of each culture was plated on MH agar plate and incubated for another 24 h. The MBC of DMSN@OL was determined based on the presence or absence of bacterial colony.

***Bactericidal effect of DMSN@OL.*** For live/dead staining, bacterial suspension, either NC or SCV, was treated with DMSN@OL at a concentration of 1200 μg mL^-1^ in confocal dishes for 24 h. After removing the spent medium and washing with PBS, the bacteria were stained using the LIVE/DEAD BacLight Viability Kit and subsequently observed using CLSM (Leica, TCS SP8, German).

For TEM analysis, following the aforementioned treatment, bacterial cells were harvested by scraping and subsequently fixed with 2.5% glutaraldehyde. Subsequently, the cells underwent dehydration through a gradient series of ethanol solutions and were embedded in resin to enable ultrathin sectioning. The resulting ultrathin sections were stained with uranyl acetate and examined using a TEM.

For RNA-seq and metabolomics analyses (SCV + DMSN@OL *vs.* SCV), the experimental method has been previously described.

***Biosafety analysis.*** MC3T3-E1 cells were seeded at a density of 1 × 10^5^ cells per well in a 24-well plate and cultured overnight at 37°C with 5% CO_2_. To evaluated cell viability, 1200 μg mL^-1^ of DMSN@OL or an equivalent concentration of Oxa (217.56 μg mL^-1^) and Lon (338.76 μg mL^-1^) were added to the cells. Cell viability was subsequently assessed on days 1, 2, 4 using the CCK-8 assay kit (Dojindo, Kumamoto, Japan) in accordance with the manufacturer’s instructions. To measure ATP content, 1200 μg/mL of DMSN@Lon or an equivalent concentration of Lon (338.76 μg/mL) was added to the cells. ATP content was then measured using ATP assay kits (Beyotime Biotechnology, Shanghai, China) on days 1, 2, and 4. To prepare the DMSN-OL extract, DMSN@OL was dispersed in α-MEM complete medium and adjusted to a concentration of 1200 μg/mL. The dispersion was then adjusted to pH 6.0 and 7.4, respectively, and incubated at 37°C for 15 days. After centrifugation, the supernatant was collected for culturing MC3T3-E1 cells, and cell viability was assessed after 1 day using the CCK-8 assay kit.

For RT-PCR analysis of ALP, RUNX2, and OCN, MC3T3-E1 cells were seeded at a density of 1 × 10^5^ cells per well in a 24-well plate and cultured for 1 day. The medium was then replaced with osteogenic induction medium (α-MEM complete medium supplemented with 50 μg/mL of ascorbic acid, 10 mmol/L of β-glycerophosphate, and 0.1 μmol/L of dexamethasone) containing DMSN@Lon (1200 μg/mL) or Lon (338.76 μg/mL) for continued culture over two weeks. Cell samples were subsequently lysed, and total RNA was extracted using the RNA Purification Kit (EZBionscience). The remaining steps were performed as previously described. The primers for ALP, RUNX2, and OCN are listed in the Table S8.

***Evaluation of in vivo therapeutic efficacy of DMSN@OL.*** Sixty Balb/c mice (male, 6-8 weeks) were evenly divided, and 10 µL of 1 × 10^8^ CFU/mL NC or SCV suspension was used to construct osteomyelitis models on day -3 according to the method described before. Additionally, six mice in each model were not inoculated with bacteria and were designated as no bacteria (NB) group. After MRI confirmed the success of the modeling (day -1), the wounds of the infected mice were reopened and equal volumes (10 µL) of DMSN@OL (6000 μg mL^-1^), Oxa (1087.8 μg mL^-1^), a mixed solution of Oxa and Lon (1087.8 μg mL^-1^ and 1693.8 μg mL^-1^, respectively), and PBS were injected into the bone marrow cavity according to group allocation using a microsyringe. After closing the incision, 40 μL of the corresponding preparation was injected into the infection foci of soft tissue. The same treatment was repeated on day 4 and day 9. Every three days, the body weight of the mice was measured, as well as the circumference of the knee joint. On day 10 and day 30, small amounts of blood samples were collected from the retro-orbital venous plexus for the hematological analysis.

On day 30, the mice were scanned using MRI as described before, then euthanized, and the infected legs were extracted for observation and photography. A small amount of infected soft tissue around the knee joint was collected, weighed, ground, and serially diluted for standard plate counting to evaluate the bacterial load. For micro-CT analysis, the samples were scanned using a micro-CT scanner (SkyScan 1176, Bruker, Germany) with a layer thickness of 9 μm. The obtained images were reconstructed using Mimics 21.0 software (Materialise, Leuven, Belgium) and quantitatively analyzed using CTAn software (Skyscan v.1.17.7.2).

The samples were fixed, decalcified, dehydrated, embedded, and sectioned according to the protocol outlined before. The sections were then subjected to HE, Gram, Masson, and TRAP staining, followed by examination by microscopy. For immunofluorescence staining, the sections were incubated overnight at 4°C in the dark with anti-mouse TGF-β antibody (1:100, Sigma) or anti-mouse TNF-α antibody (1:500, Abcam). They were then stained with Alexa Fluor 594 conjugated anti-rabbit antibody (1:500, Abcam) for 2 h before observation. Semi-quantitative analysis of Masson and immunofluorescence staining was performed using ImageJ (version 1.48v, NIH, USA). Additionally, sections of the main organs (heart, liver, spleen, lungs, and kidneys) were also prepared and stained with HE for biosafety analysis.

***Statistical analysis.*** All statistical metrics included data from at least three independent samples, presented as mean ± standard deviation, with statistical differences analyzed using Origin 2022 (OriginLab, MA, USA) and GraphPad Prism v9.0.0 (La Jolla, CA, USA). The primary statistical methods used were one-way ANOVA and Student’s t-test. The correlation between infection course and small colonies was calculated by point-biserial correlation analysis. Statistical significance was denoted as * (p < 0.05), ** (p < 0.01), *** (p < 0.001), and **** (p < 0.0001).

**Supplementary Figures**


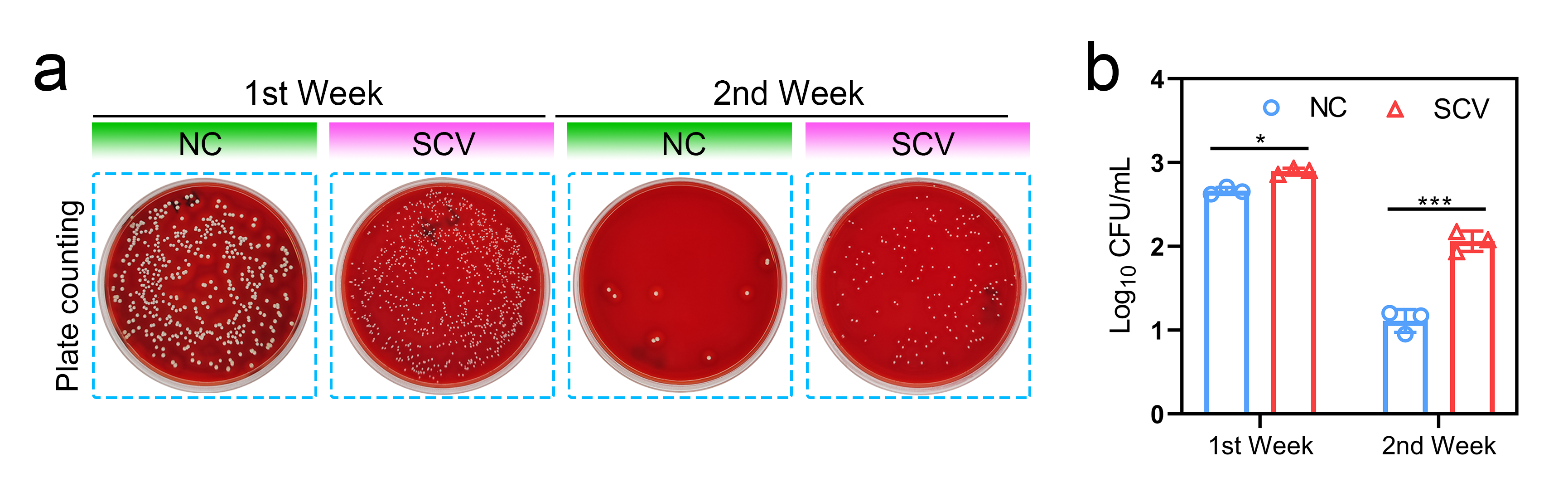


**Figure S1.** (a) Standard plate counting test assessing remaining bacterial loads in bone marrow. (b) Statistical analysis of bacterial colonies. Results are presented as the mean ± SD, with n = 3.


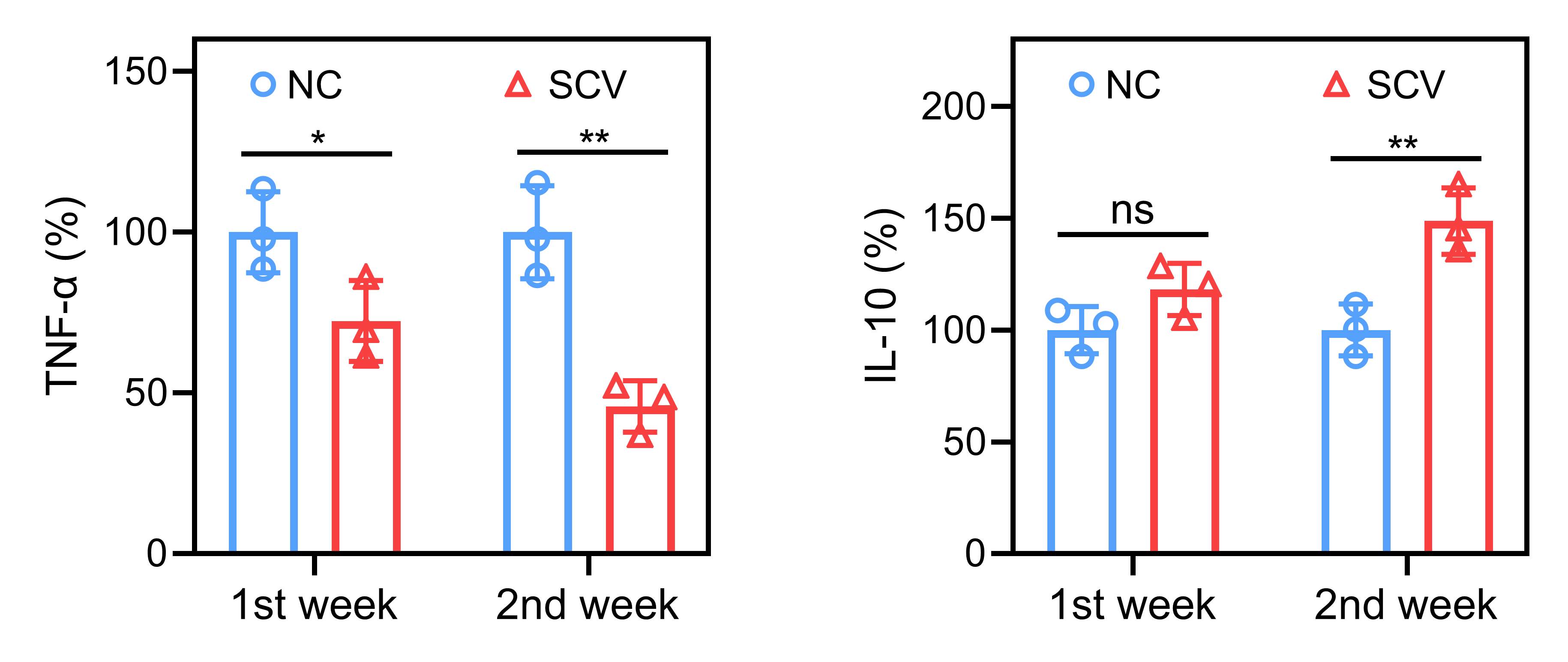


**Figure S2.** Determination of TNF-α and IL-10 levels in bone marrow fluid using ELISA. Results are presented as the mean ± SD, with n = 3.


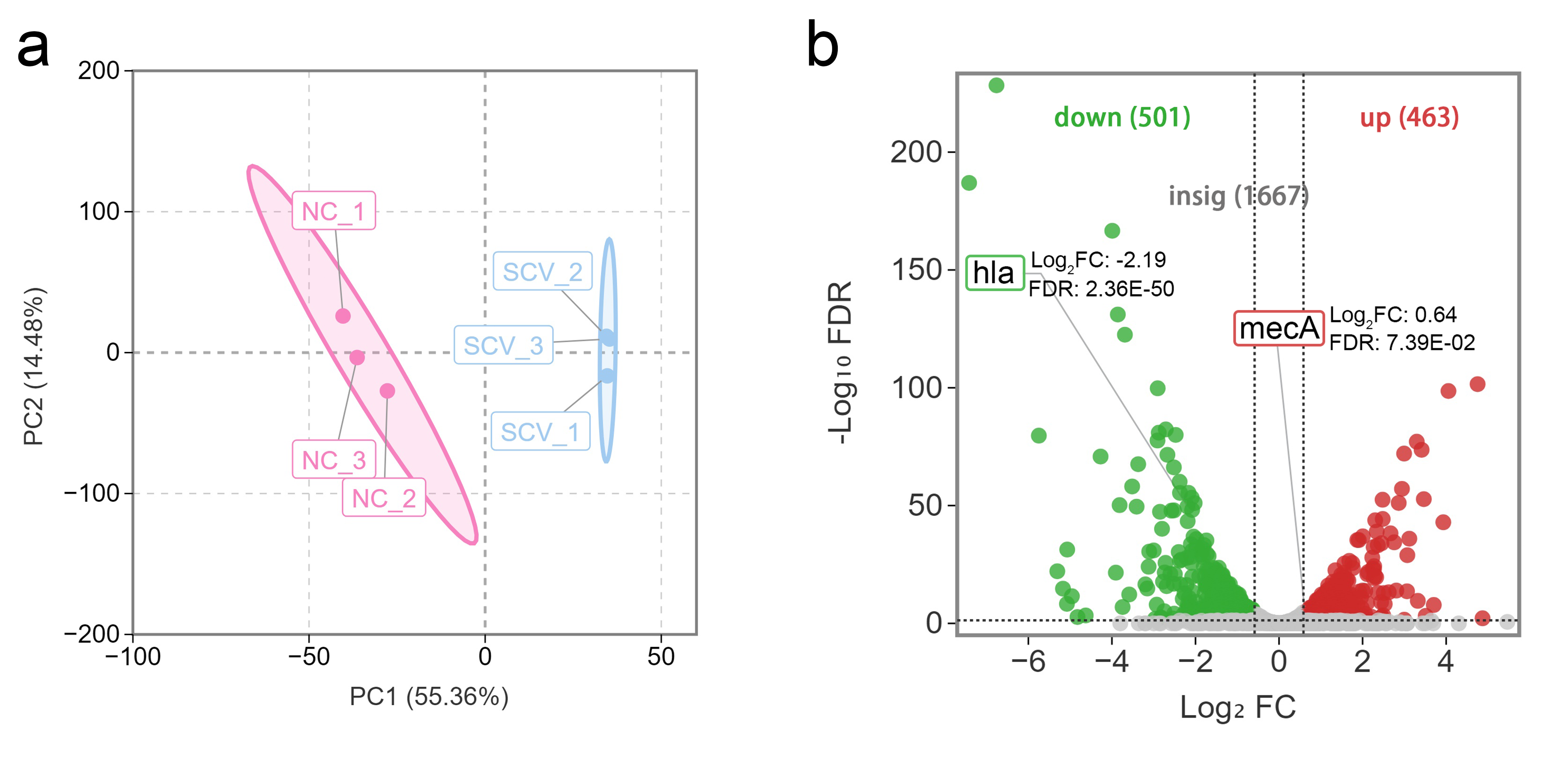


**Figure S3.** (a) Principal component analysis based on gene expression level of NC and SCV group. (b) Volcano plot of DEGs.


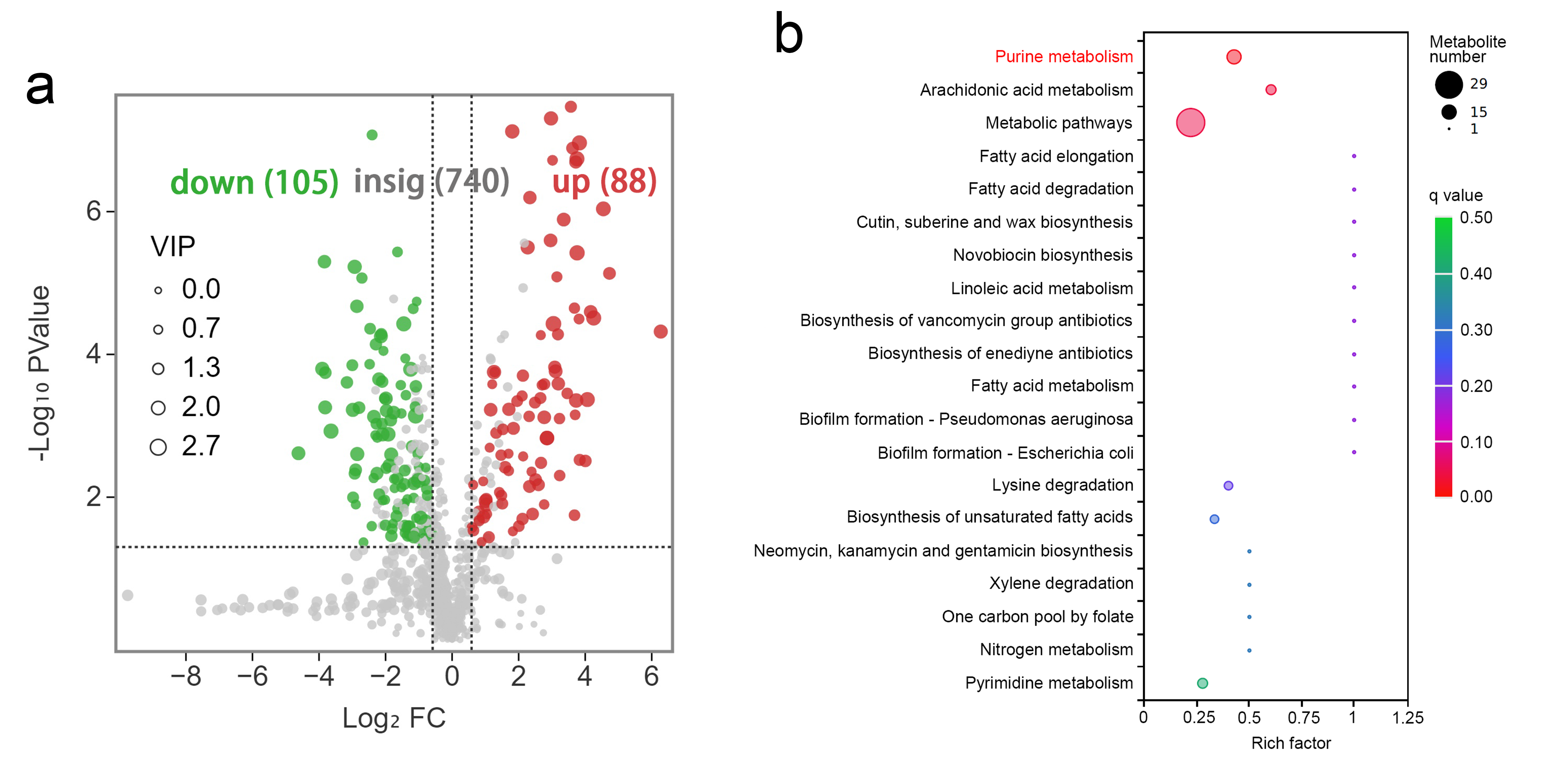


**Figure S4.** (a) Volcano plot displaying DEMs between NC and SCV groups. (b) Top 20 KEGG pathways based on DEMs between NC and SCV.


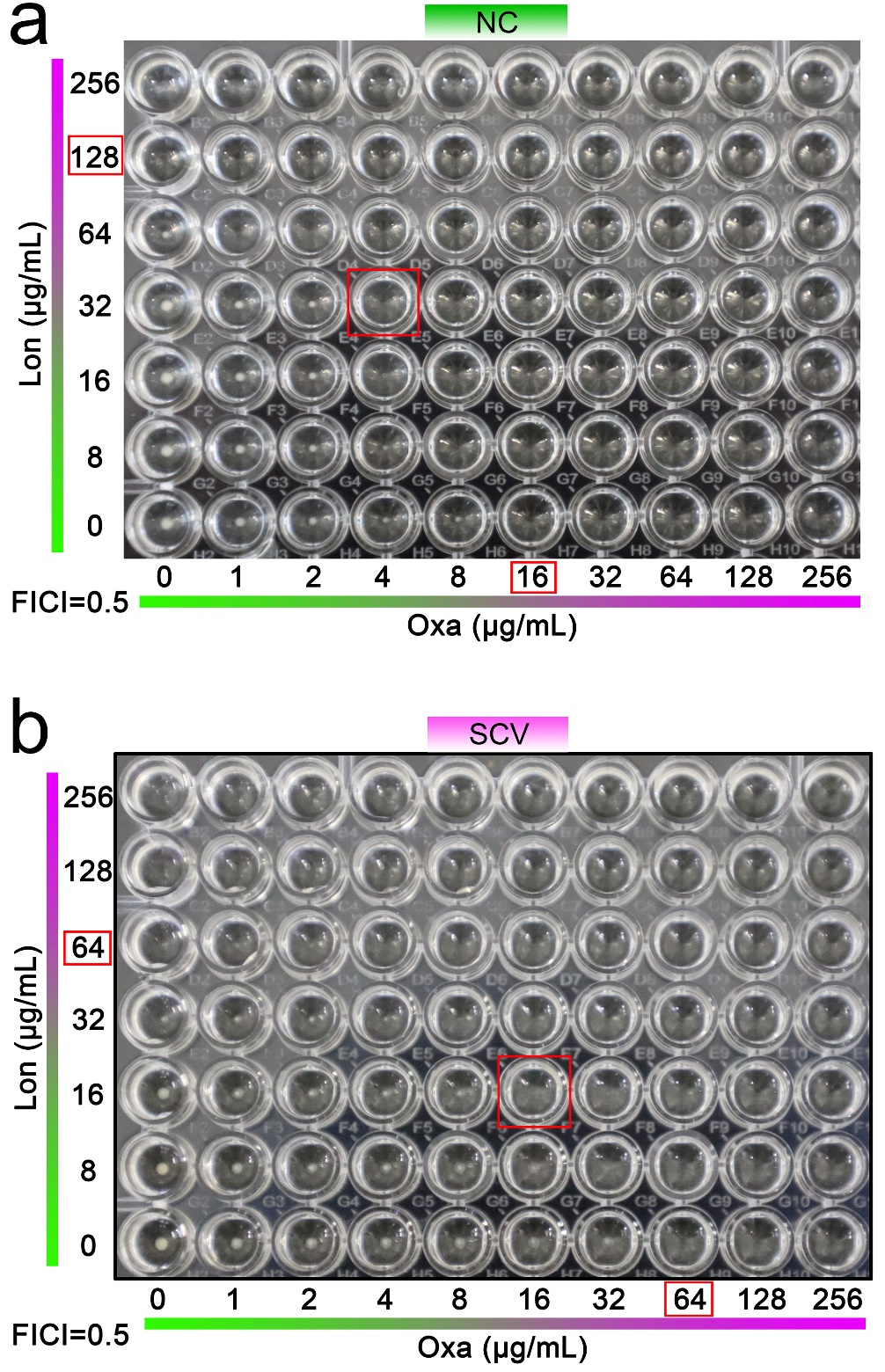


**Figure S5.** Checkerboard broth microdilution assay evaluating the synergistic effect of Lon and Oxa against NC (a) or SCV (b). NC growth was completely inhibited by 4 µg/mL Oxa combined with 32 µg/mL Lon, while SCV growth was completely inhibited by 16 µg/mL Oxa combined with 16 µg/mL Lon.


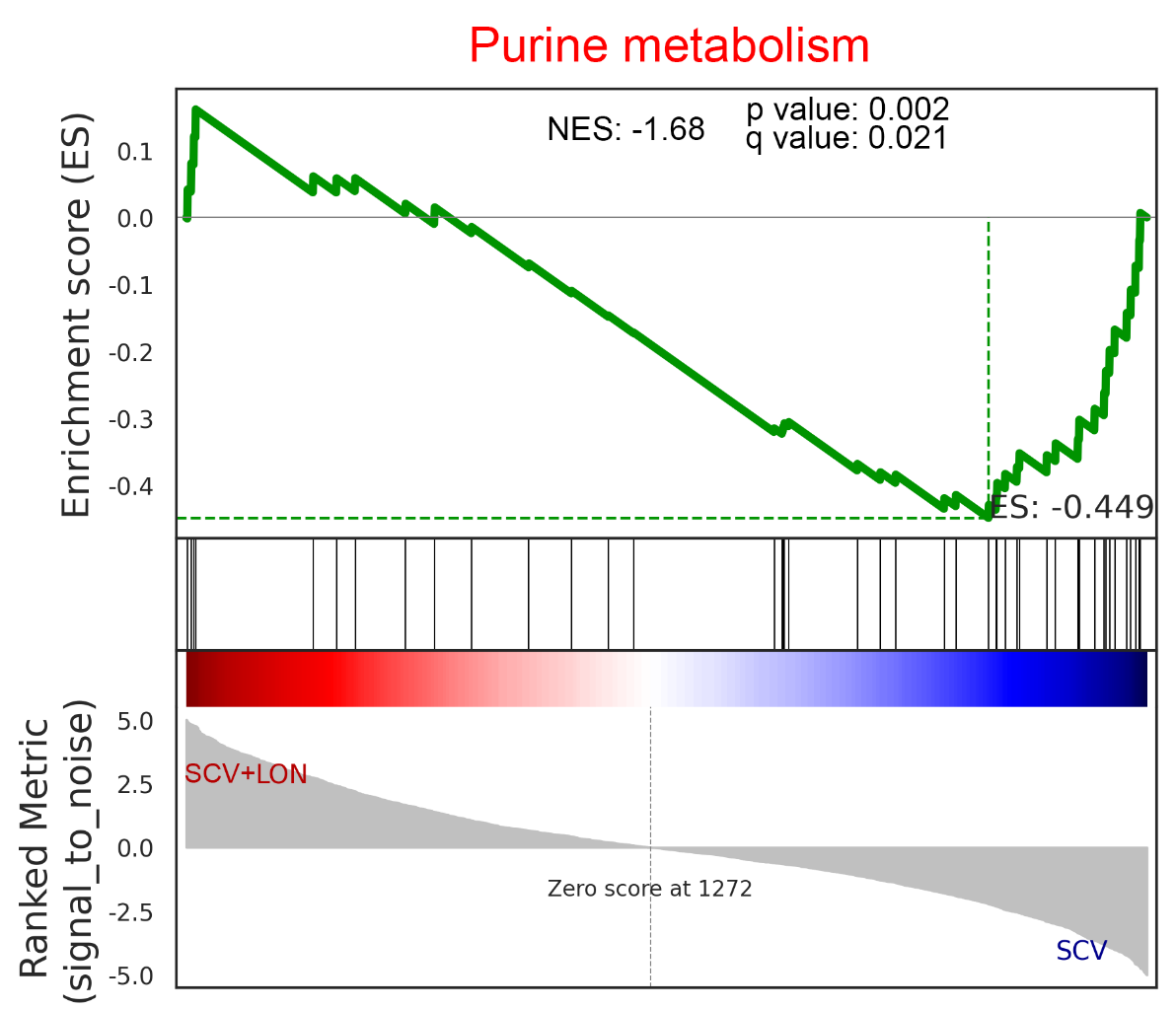


**Figure S6.** GSEA of purine metabolism pathway in SCV after treated by Lon.


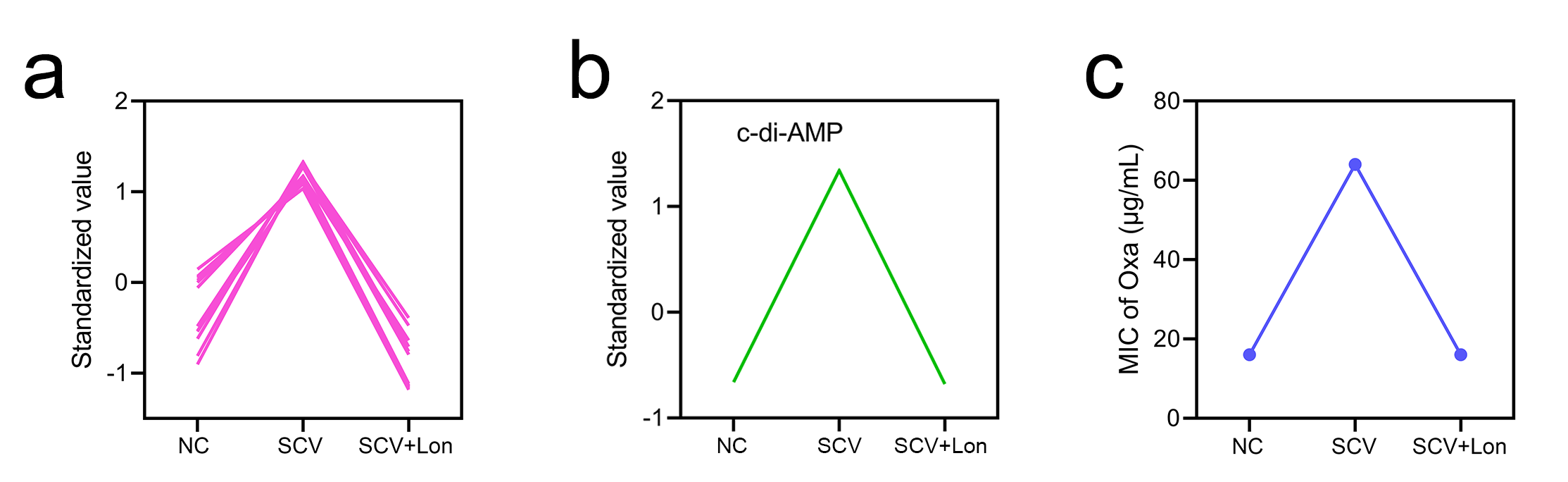


**Figure S7.** Change Patterns of (a) shared DEGs’ expression, (b) c-di-AMP levels, and (c) MIC values among NC group, SCV group, and SCV + Lon group. Shared DEGs are defined as DEGs in both SCV *vs.* NC and SCV + Lon *vs.* SCV comparisons.


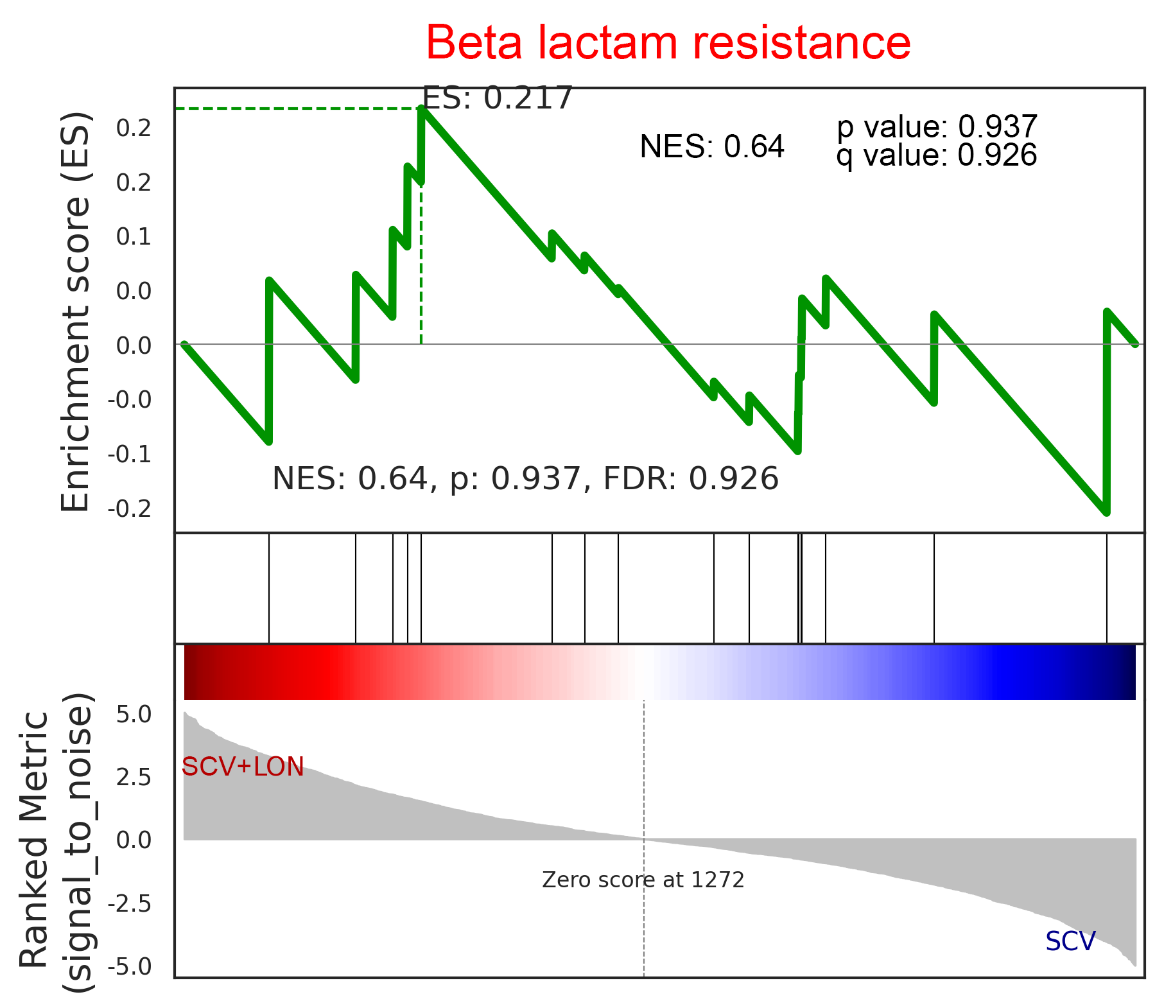


**Figure S8.** GSEA of β-lactam resistance pathway in SCV after treated by Lon.


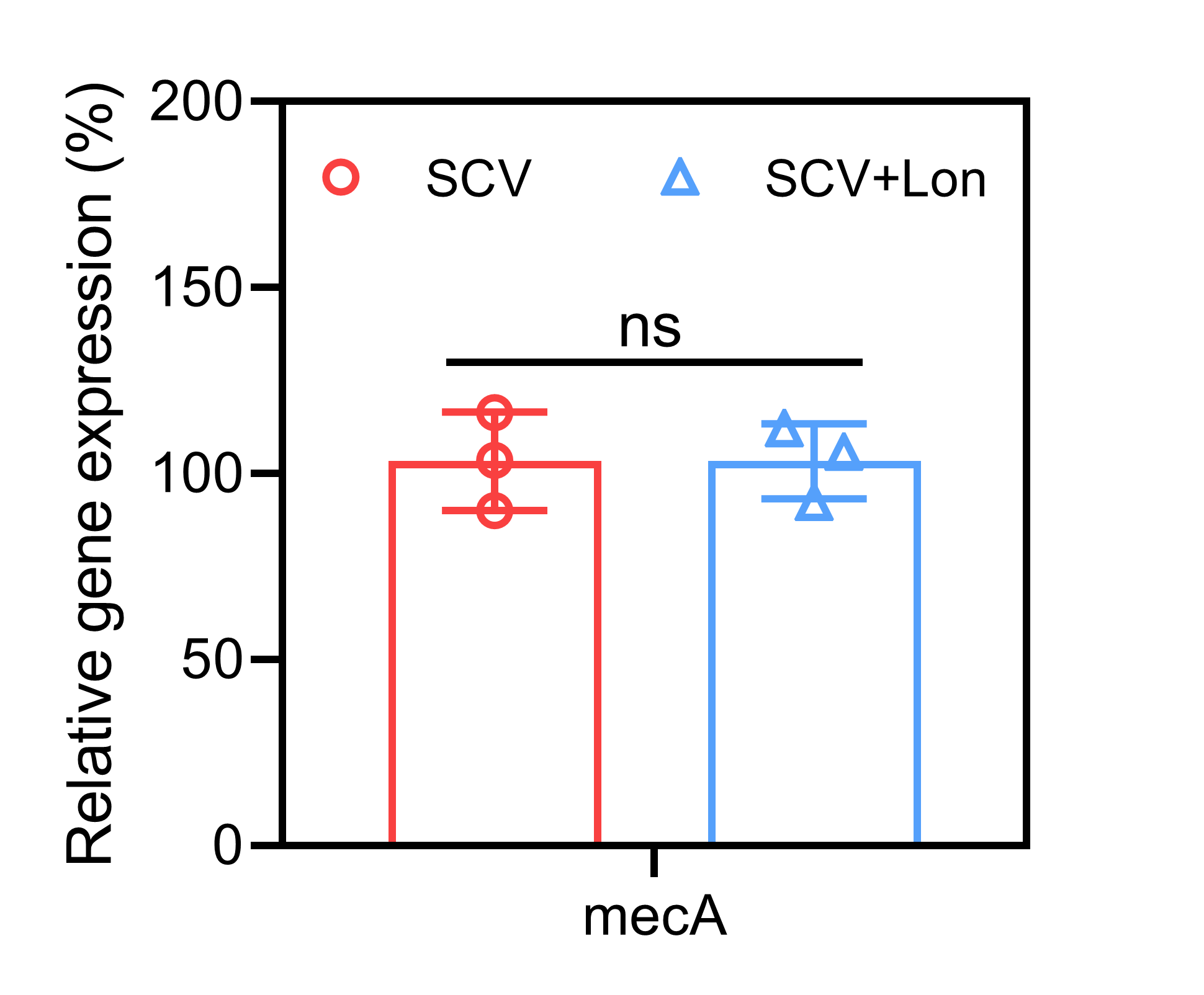


**Figure S9.** RT-PCR analysis of *mecA* expression. Results are presented as the mean ± SD, with n = 3.


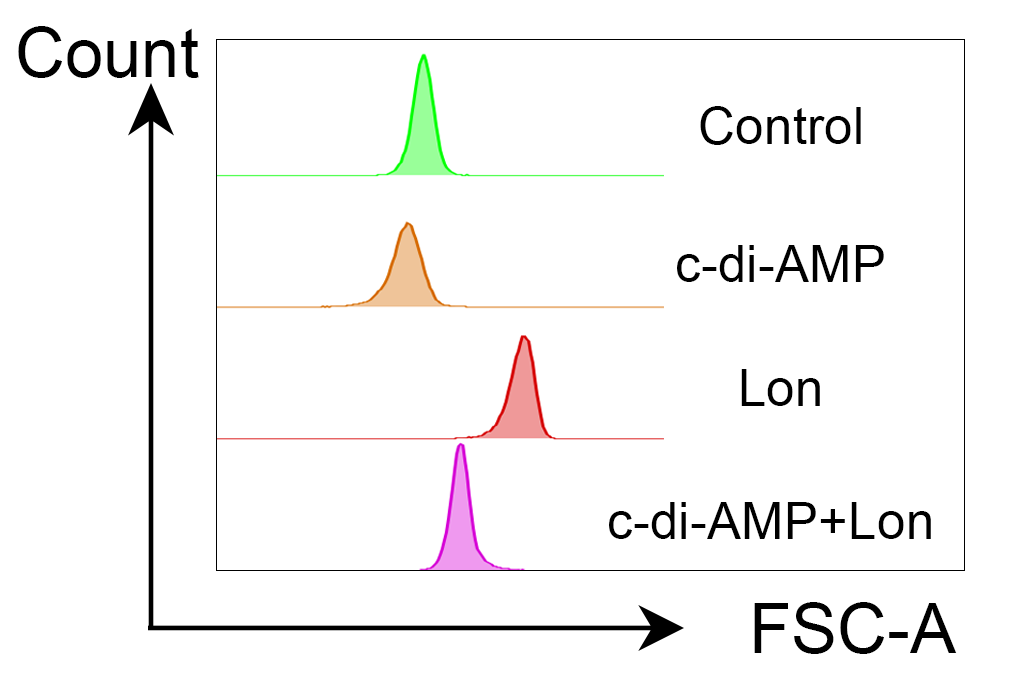


**Figure S10.** Flow cytometry analysis showing the FSC-A of SCV following treatment.


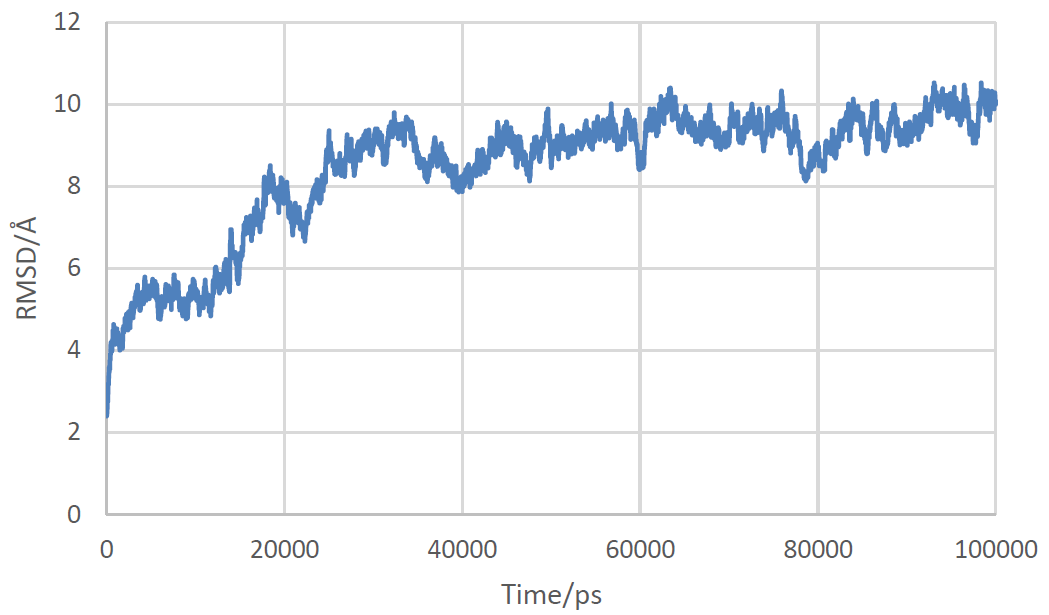


**Figure S11.** Root mean square deviation (RMSD) analysis of the dacA protein following its interaction with Lon.

**
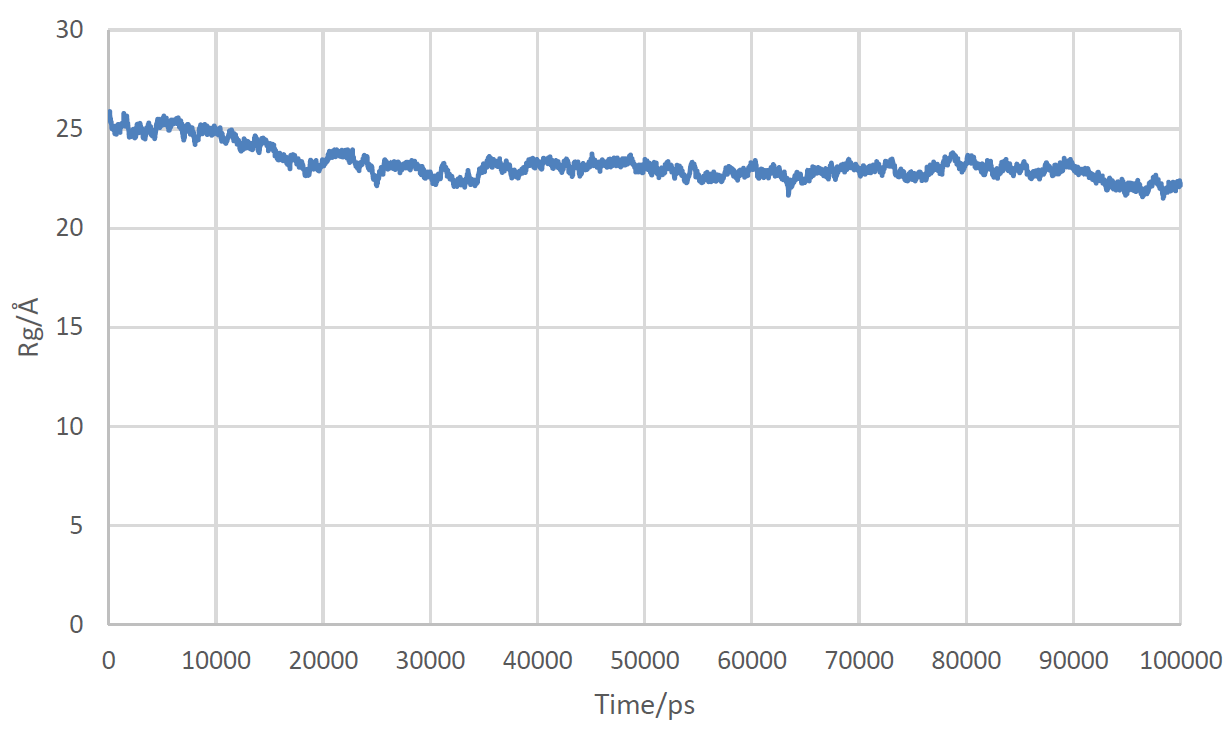
**

**Figure S12.** Radius of gyration (Rg) analysis of the dacA protein following its interaction with Lon


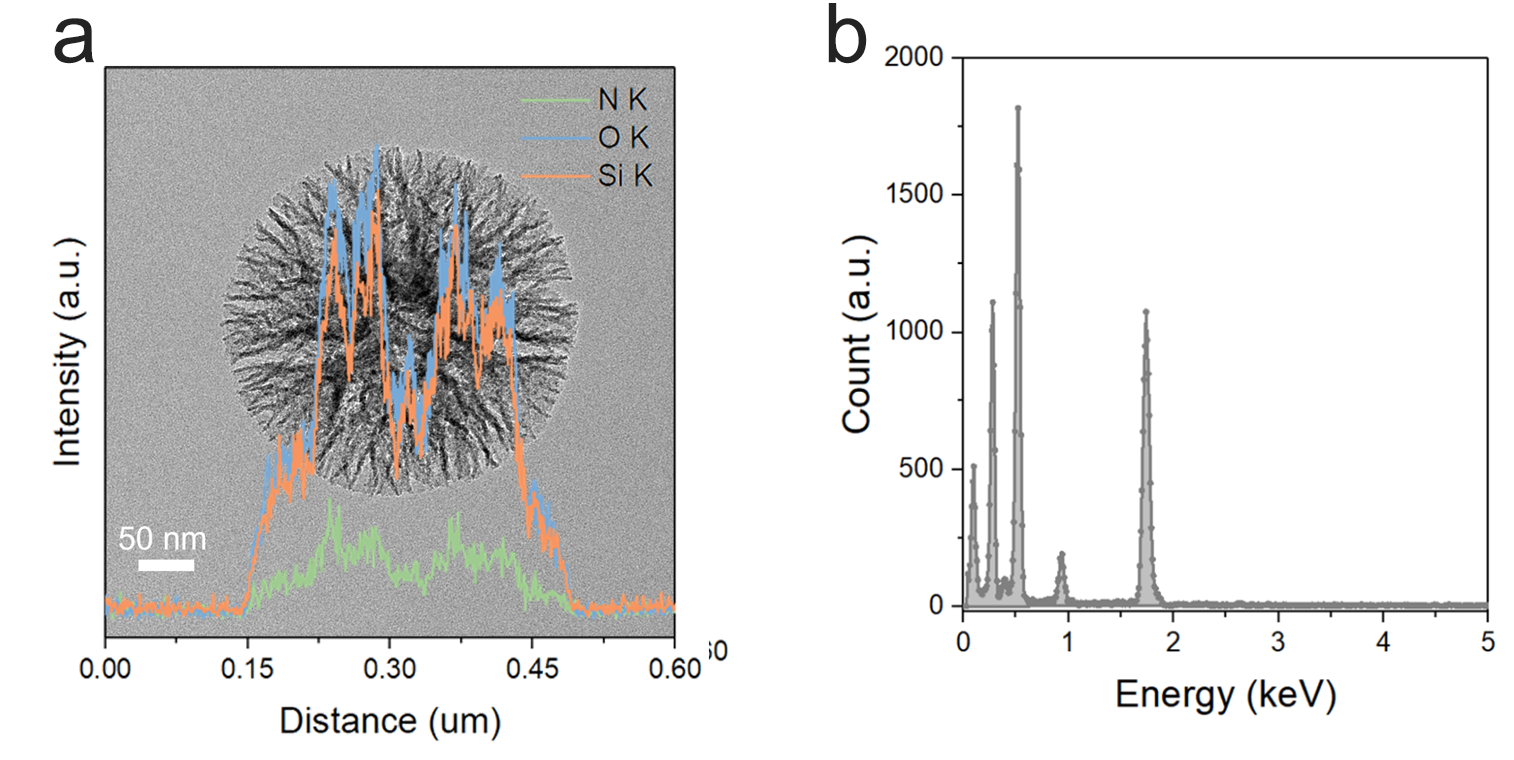


**Figure S13.** (a) Linear scan element distribution of a single DMSN nanoparticle and (b) the EDS spectrum.


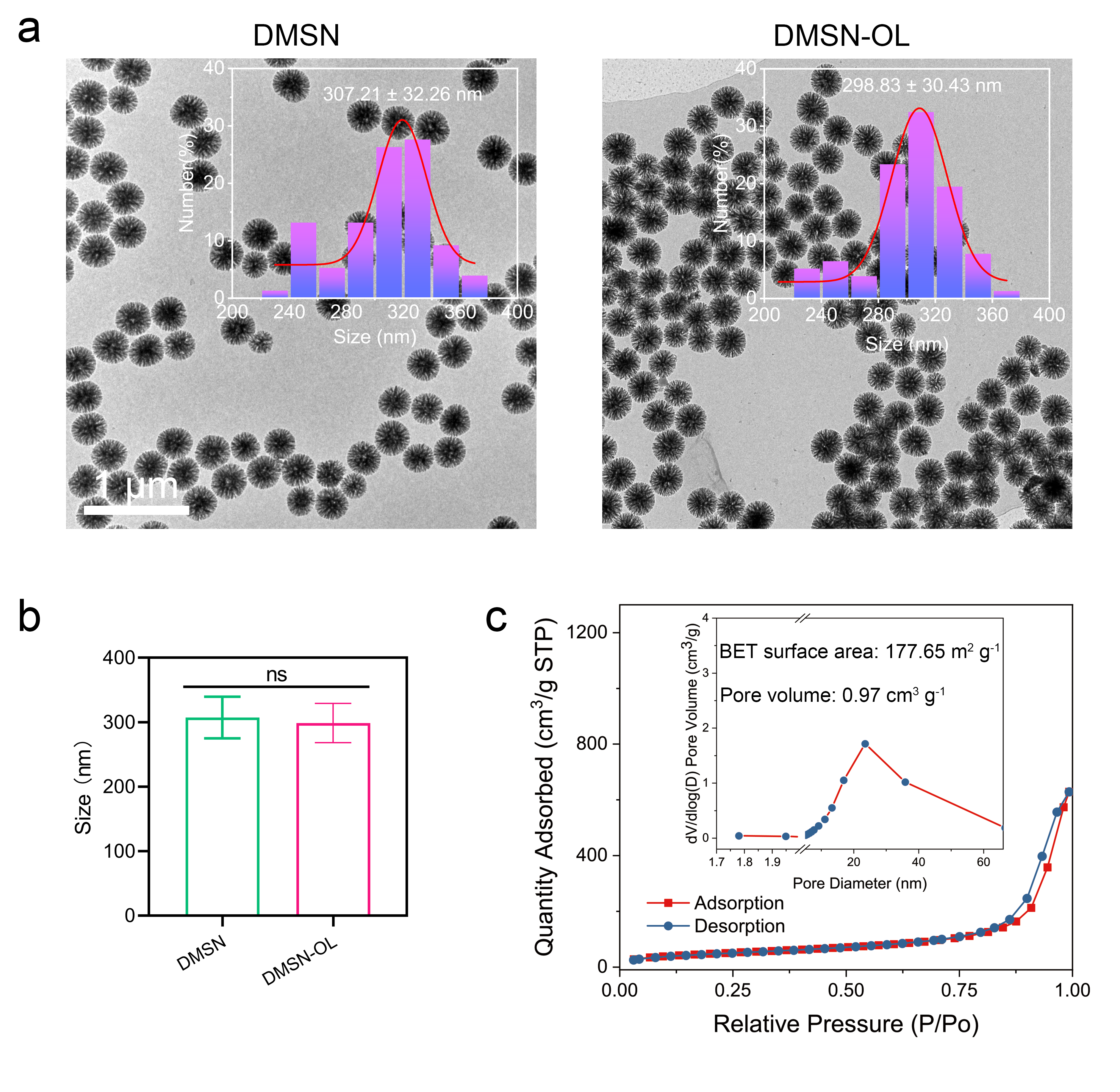


**Figure S14.** (a) TEM images and particle size distribution curves of DMSN and DMSN-OL. (b) Statistical analysis of particle size differences between DMSN and DMSN-OL (The particle sizes were measured by Nano Measurer software). (c) N₂ adsorption-desorption isotherms of DMSN-OL.


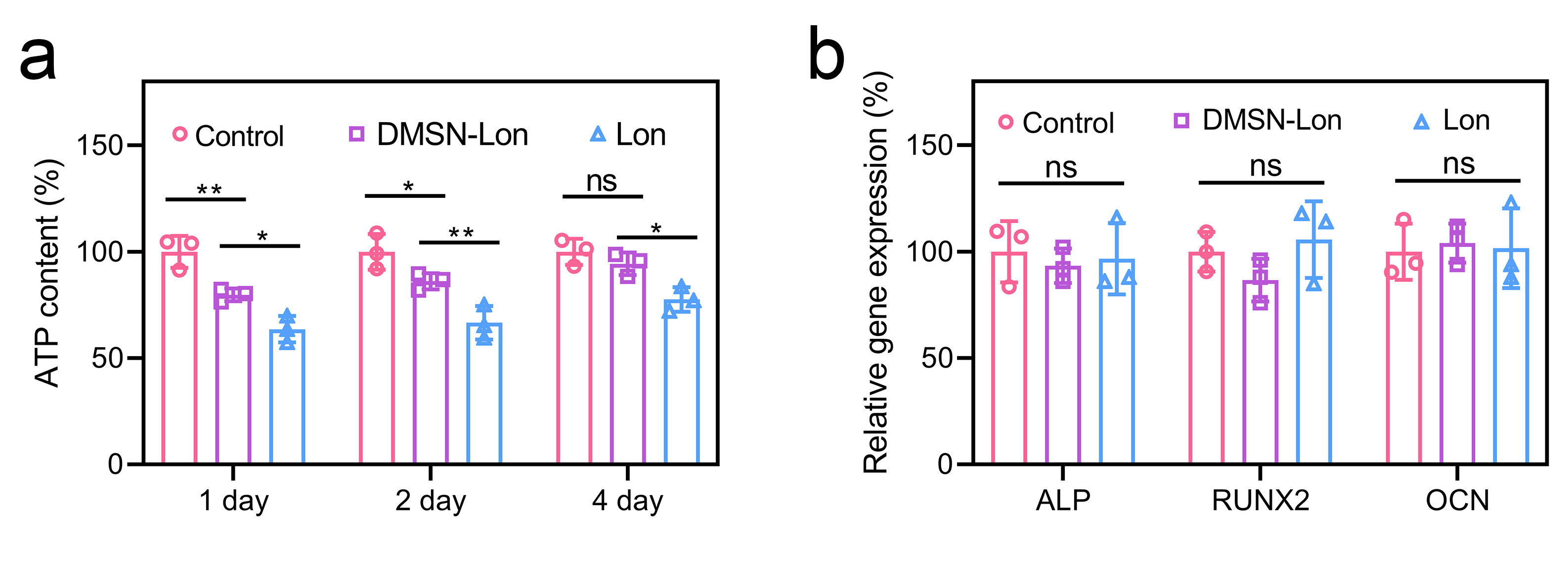


**Figure S15.** (a) ATP content measurement in MC3T3-E1 cells. (b) Relative gene expression of ALP, RUNX2, and OCN using RT-PCR. Results are presented as the mean ± SD, with n = 3.


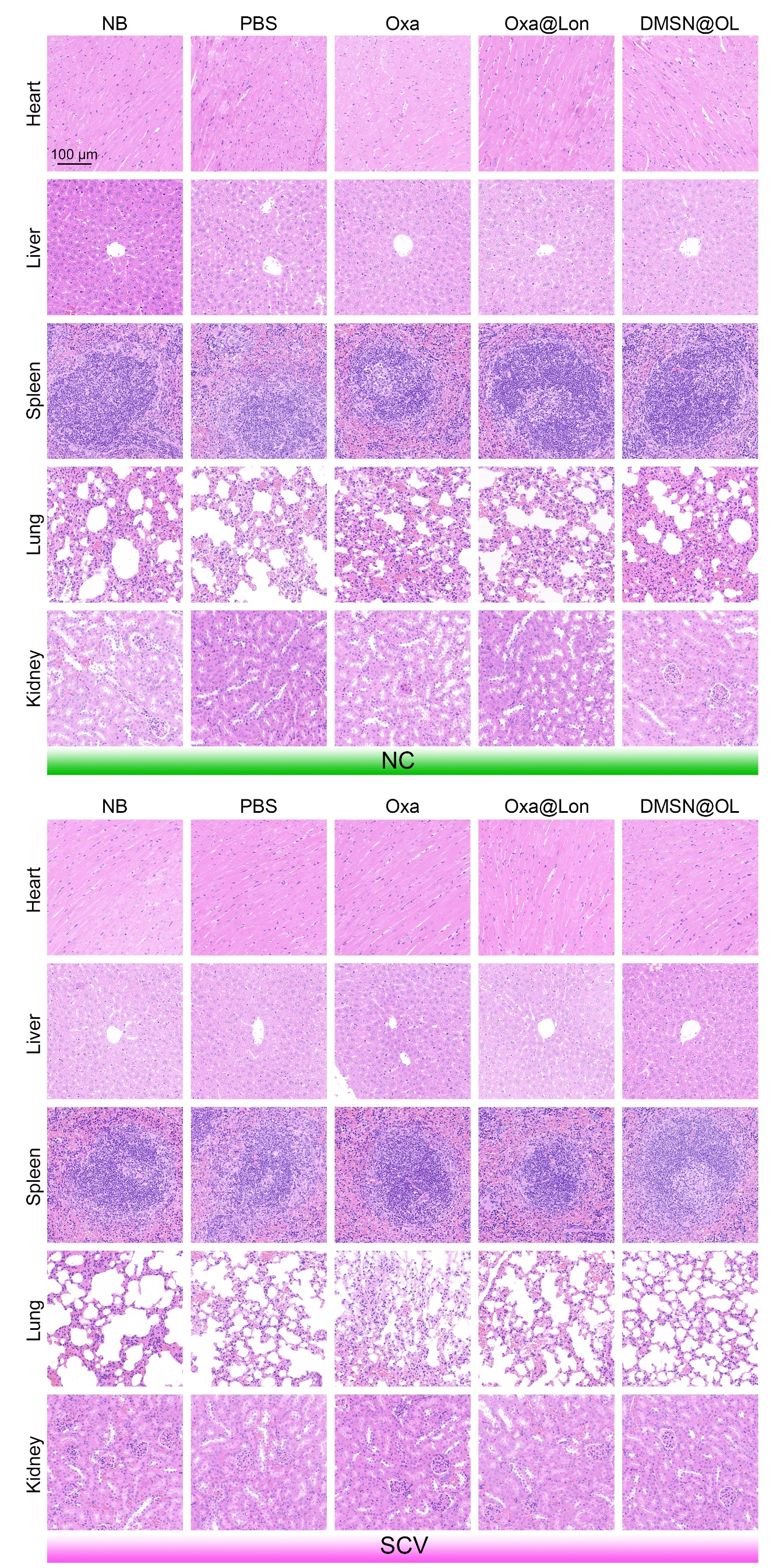


**Figure S16.** HE staining of histological sections of major organs (heart, liver, spleen, lungs, kidneys).


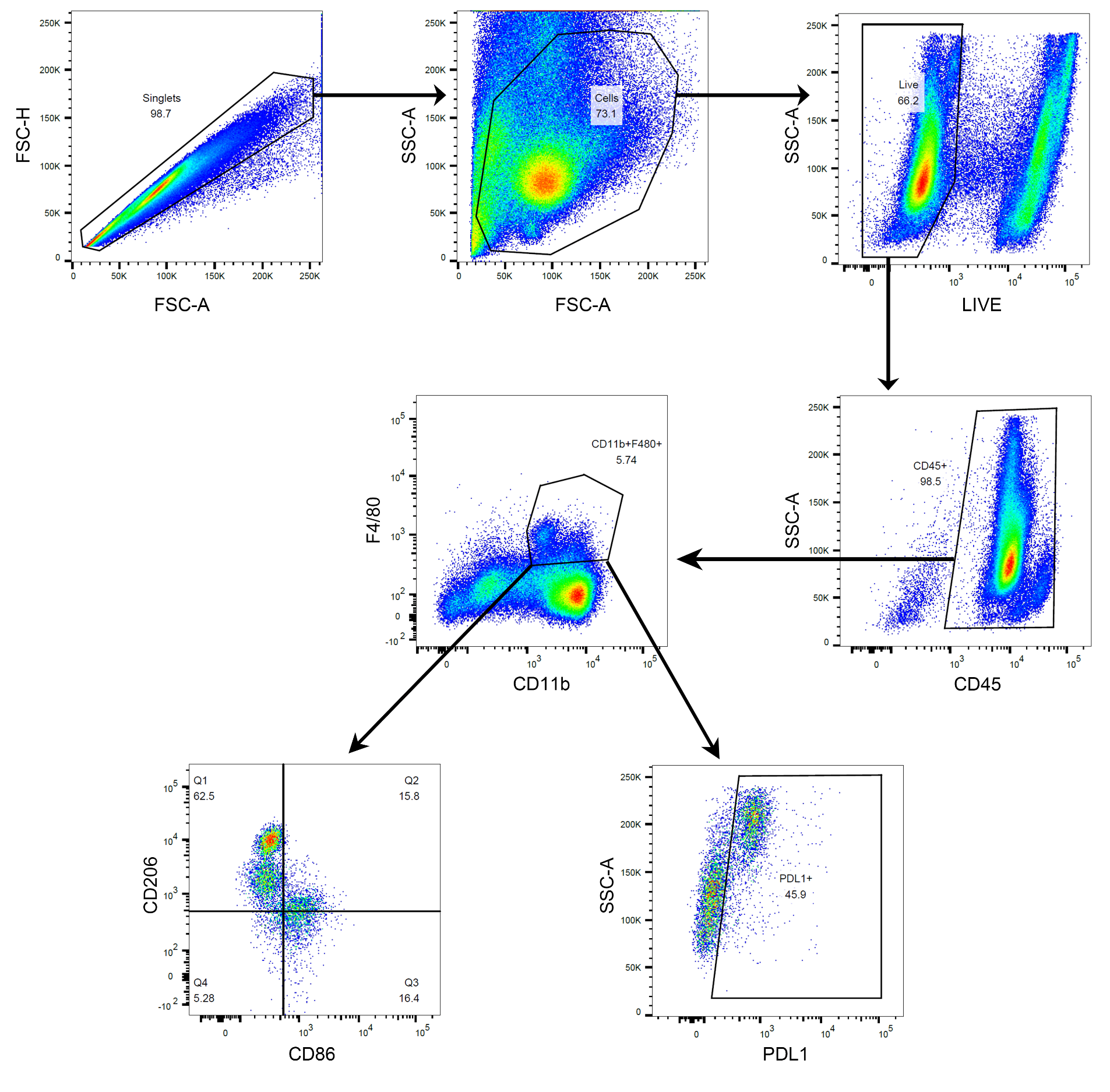


**Figure. S17.** Flow cytometric gating strategy used to determine CD206^+^CD86^-^ macrophages and PDL1^+^ macrophages in the bone marrow.


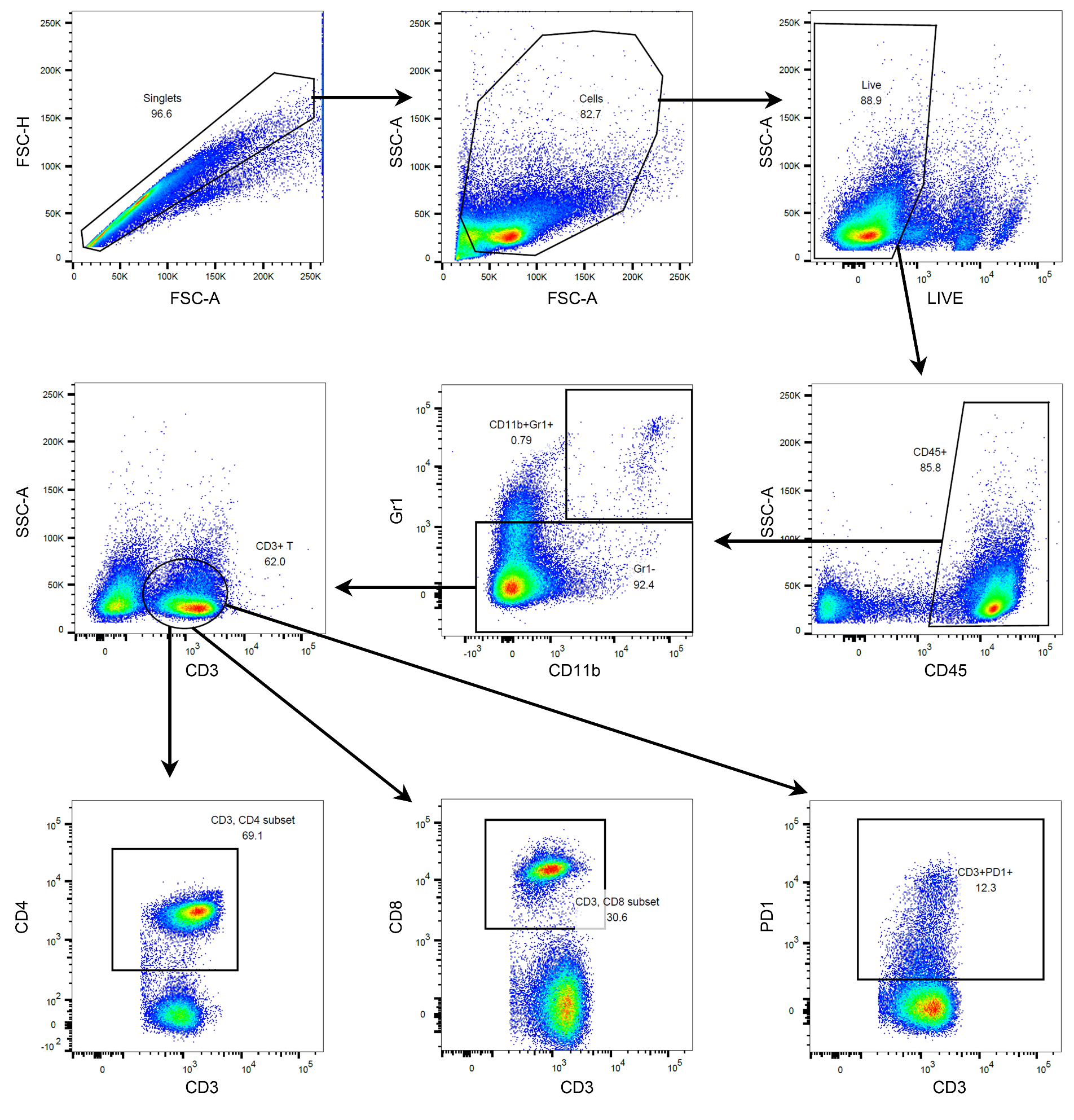


**Figure S18.** Flow cytometric Gating strategy used to determine CD4^+^ T cells, CD8^+^ T cells and PD1^+^ T cells in infection-draining lymph nodes.

**Tab****le S1.** Nonsynonymous gene mutations exclusive to clinically-isolated stable SCV strains.

|  | **Gene** | **Product** | **type** | **Amino acid change** |
| --- | --- | --- | --- | --- |
| Ⅰ | *hpt* | hypoxanthine phosphoribosyltransferase | SNP | Gly43Cys |
| II | *clpX* | ATP-dependent Clp protease ATP-binding subunit ClpX | SNP | Met233Lys |
|  | *ptsG* | glucose-specific PTS transporter subunit IIBC | SNP | Ala451Thr |
|  | *trmL* | tRNA (uridine(34)/cytosine(34)/5- carboxymethylaminomethyluridine(34)-2'-O)- methyltransferase TrmL | SNP | Arg131His |
|  | *hpt* | hypoxanthine phosphoribosyltransferase | DEL | Phe151fs |
| III | *sbi* | immunoglobulin-binding protein Sbi | INS | Glu290/Val291insValProGlnIleGlnSerProLysValGlu |
|  | *hpt* | hypoxanthine phosphoribosyltransferase | DEL | Ile147fs |
|  | *IsdE* | High-affinity heme uptake system protein IsdE | DEL | Ala74fs |
| IV | *aroC* | chorismate synthase | SNP | Gly111Arg |
|  | *infB* | translation initiation factor IF-2 | SNP | Lys55Asn |
|  | *hpt* | hypoxanthine phosphoribosyltransferase | SNP | Ala45Glu |
|  | *IsdE* | High-affinity heme uptake system protein IsdE | SNP | Ser111Ile |
| V | *yycF* | response regulator YycF | SNP | Thr201Met |
|  | *stp* | protein-serine/threonine phosphatase Stp1 | DEL | Tyr186fs |
|  | *IsdC* | Iron-regulated surface determinant protein C | SNP | Ile117Val |

**Table S2.** Gene set enrichment analysis (GSEA) of the KEGG pathways based on DEGs between NC group and SCV group.

| **Term order** | **Term name** | **ES** | **NES** | **p value** | **q value** | **Regulation** |
| --- | --- | --- | --- | --- | --- | --- |
| 1 | Ribosome | 0.71 | 2.88 | 0.00 | 0.00 | up |
| 2 | Pyrimidine metabolism | 0.49 | 1.65 | 0.01 | 0.07 | up |
| 3 | Purine metabolism | 0.43 | 1.62 | 0.00 | 0.07 | up |
| 4 | Galactose metabolism | 0.53 | 1.56 | 0.03 | 0.08 | up |
| 5 | Mismatch repair | 0.49 | 1.51 | 0.05 | 0.10 | up |
| 6 | Protein export | 0.52 | 1.51 | 0.04 | 0.08 | up |
| 7 | Aminoacyl tRNA biosynthesis | 0.45 | 1.48 | 0.04 | 0.09 | up |
| 8 | DNA replication | 0.50 | 1.46 | 0.07 | 0.08 | up |
| 9 | Homologous recombination | 0.44 | 1.40 | 0.08 | 0.11 | up |
| 10 | Biosynthesis of cofactors | 0.28 | 1.25 | 0.07 | 0.21 | up |
| 11 | Nucleotide metabolism | 0.35 | 1.24 | 0.15 | 0.21 | up |
| 12 | Glyoxylate and dicarboxylate metabolism | 0.32 | 1.05 | 0.37 | 0.46 | up |
| 13 | Glycerophospholipid metabolism | 0.26 | 0.81 | 0.74 | 0.89 | up |
| 14 | Teichoic acid biosynthesis | 0.21 | 0.66 | 0.93 | 1.00 | up |
| 15 | Fatty acid metabolism | 0.21 | 0.63 | 0.95 | 0.96 | up |
| 16 | Peptidoglycan biosynthesis | 0.17 | 0.57 | 0.98 | 0.92 | up |
| 17 | Citrate cycle (TCA cycle) | -0.18 | -0.50 | 0.99 | 0.96 | down |
| 18 | Glycerolipid metabolism | -0.28 | -0.76 | 0.79 | 0.84 | down |
| 19 | Oxidative phosphorylation | -0.28 | -0.81 | 0.73 | 0.79 | down |
| 20 | Quorum sensing | -0.25 | -0.87 | 0.67 | 0.71 | down |
| 21 | Beta-lactam resistance | -0.33 | -0.93 | 0.57 | 0.64 | down |
| 22 | Carbon metabolism | -0.25 | -0.95 | 0.56 | 0.62 | down |
| 23 | Biosynthesis of nucleotide sugars | -0.35 | -0.99 | 0.45 | 0.56 | down |
| 24 | Arginine biosynthesis | -0.37 | -1.04 | 0.41 | 0.50 | down |
| 25 | Amino sugar and nucleotide sugar metabolism | -0.37 | -1.15 | 0.26 | 0.33 | down |
| 26 | Folate biosynthesis | -0.43 | -1.23 | 0.19 | 0.24 | down |
| 27 | Starch and sucrose metabolism | -0.48 | -1.28 | 0.17 | 0.19 | down |
| 28 | Porphyrin metabolism | -0.48 | -1.30 | 0.14 | 0.19 | down |
| 29 | Glycolysis gluconeogenesis | -0.43 | -1.38 | 0.08 | 0.12 | down |
| 30 | Glycine serine and threonine metabolism | -0.45 | -1.40 | 0.08 | 0.11 | down |
| 31 | Methane metabolism | -0.52 | -1.41 | 0.07 | 0.11 | down |
| 32 | Cysteine and methionine metabolism | -0.48 | -1.43 | 0.07 | 0.10 | down |
| 33 | Pyruvate metabolism | -0.43 | -1.45 | 0.04 | 0.09 | down |
| 34 | Phenylalanine tyrosine and tryptophan biosynthesis | -0.52 | -1.46 | 0.07 | 0.09 | down |
| 35 | Propanoate metabolism | -0.49 | -1.48 | 0.06 | 0.08 | down |
| 36 | Alanine aspartate and glutamate metabolism | -0.54 | -1.53 | 0.03 | 0.06 | down |
| 37 | Biosynthesis of secondary metabolites | -0.37 | -1.58 | 0.00 | 0.04 | down |
| 38 | Pentose phosphate pathway | -0.54 | -1.60 | 0.02 | 0.04 | down |
| 39 | Phosphotransferase system (PTS) | -0.53 | -1.61 | 0.01 | 0.04 | down |
| 40 | Fructose and mannose metabolism | -0.61 | -1.66 | 0.02 | 0.03 | down |
| 41 | Pantothenate and CoA biosynthesis | -0.60 | -1.71 | 0.01 | 0.02 | down |
| 42 | ABC transporters | -0.46 | -1.72 | 0.00 | 0.02 | down |
| 43 | Butanoate metabolism | -0.65 | -1.82 | 0.00 | 0.01 | down |
| 44 | Microbial metabolism in diverse environments | -0.45 | -1.83 | 0.00 | 0.01 | down |
| 45 | *Staphylococcus aureus* infection | -0.62 | -1.92 | 0.00 | 0.00 | down |
| 46 | Two component system | -0.55 | -2.02 | 0.00 | 0.00 | down |
| 47 | 2 oxocarboxylic acid metabolism | -0.80 | -2.23 | 0.00 | 0.00 | down |
| 48 | Biosynthesis of amino acids | -0.60 | -2.32 | 0.00 | 0.00 | down |

**Table S3.** GSEA analysis of the KEGG pathways based on DEGs between SCV group and SCV + Lon group.

| **Term order** | **Term name** | **ES** | **NES** | **p value** | **q value** | **Regulation** |
| --- | --- | --- | --- | --- | --- | --- |
| 1 | Phosphotransferase system (PTS) | 0.66 | 2.12 | 0.00 | 0.00 | up |
| 2 | Starch and sucrose metabolism | 0.66 | 1.87 | 0.00 | 0.01 | up |
| 3 | Fructose and mannose metabolism | 0.64 | 1.85 | 0.00 | 0.01 | up |
| 4 | 2-oxocarboxylic acid metabolism | 0.61 | 1.84 | 0.00 | 0.01 | up |
| 5 | Carbon metabolism | 0.44 | 1.81 | 0.00 | 0.01 | up |
| 6 | Folate biosynthesis | 0.58 | 1.81 | 0.00 | 0.01 | up |
| 7 | Citrate cycle (TCA cycle) | 0.54 | 1.73 | 0.02 | 0.02 | up |
| 8 | Microbial metabolism in diverse environments | 0.39 | 1.73 | 0.00 | 0.02 | up |
| 9 | Biosynthesis of amino acids | 0.38 | 1.65 | 0.00 | 0.03 | up |
| 10 | Glycolysis gluconeogenesis | 0.42 | 1.52 | 0.02 | 0.08 | up |
| 11 | Glycine serine and threonine metabolism | 0.43 | 1.50 | 0.04 | 0.09 | up |
| 12 | ABC transporters | 0.35 | 1.46 | 0.01 | 0.10 | up |
| 13 | Pentose phosphate pathway | 0.45 | 1.41 | 0.07 | 0.13 | up |
| 14 | Methane metabolism | 0.46 | 1.37 | 0.10 | 0.15 | up |
| 15 | Fatty acid metabolism | 0.43 | 1.29 | 0.16 | 0.22 | up |
| 16 | Glyoxylate and dicarboxylate metabolism | 0.40 | 1.26 | 0.16 | 0.24 | up |
| 17 | Pantothenate and CoA biosynthesis | 0.42 | 1.26 | 0.17 | 0.23 | up |
| 18 | Butanoate metabolism | 0.42 | 1.26 | 0.17 | 0.22 | up |
| 19 | Biosynthesis of secondary metabolites | 0.25 | 1.24 | 0.04 | 0.23 | up |
| 20 | Cysteine and methionine metabolism | 0.36 | 1.21 | 0.20 | 0.26 | up |
| 21 | Propanoate metabolism | 0.35 | 1.08 | 0.35 | 0.43 | up |
| 22 | Pyruvate metabolism | 0.29 | 1.05 | 0.36 | 0.47 | up |
| 23 | Phenylalanine tyrosine and tryptophan biosynthesis | 0.31 | 0.92 | 0.60 | 0.72 | up |
| 24 | Glycerolipid metabolism | 0.30 | 0.84 | 0.71 | 0.84 | up |
| 25 | Biosynthesis of cofactors | 0.19 | 0.83 | 0.84 | 0.83 | up |
| 26 | Galactose metabolism | 0.26 | 0.76 | 0.83 | 0.92 | up |
| 27 | Amino sugar and nucleotide sugar metabolism | 0.22 | 0.72 | 0.90 | 0.93 | up |
| 28 | Biosynthesis of nucleotide sugars | 0.23 | 0.70 | 0.90 | 0.91 | up |
| 29 | Beta lactam resistance | 0.22 | 0.64 | 0.94 | 0.93 | up |
| 30 | Porphyrin metabolism | -0.29 | -0.86 | 0.67 | 0.66 | down |
| 31 | Homologous recombination | -0.27 | -0.87 | 0.67 | 0.69 | down |
| 32 | Protein export | -0.41 | -1.18 | 0.24 | 0.23 | down |
| 33 | DNA replication | -0.42 | -1.28 | 0.14 | 0.15 | down |
| 34 | Peptidoglycan biosynthesis | -0.39 | -1.30 | 0.11 | 0.14 | down |
| 35 | Oxidative phosphorylation | -0.39 | -1.30 | 0.13 | 0.15 | down |
| 36 | Mismatch repair | -0.46 | -1.42 | 0.08 | 0.08 | down |
| 37 | Aminoacyl tRNA biosynthesis | -0.44 | -1.45 | 0.05 | 0.07 | down |
| 38 | Arginine biosynthesis | -0.48 | -1.47 | 0.06 | 0.07 | down |
| 39 | Nucleotide metabolism | -0.43 | -1.50 | 0.03 | 0.06 | down |
| 40 | Ribosome | -0.42 | -1.64 | 0.00 | 0.03 | down |
| 41 | Quorum sensing | -0.43 | -1.65 | 0.01 | 0.03 | down |
| 42 | Purine metabolism | -0.45 | -1.68 | 0.00 | 0.02 | down |
| 43 | Glycerophospholipid metabolism | -0.56 | -1.70 | 0.01 | 0.02 | down |
| 44 | Pyrimidine metabolism | -0.56 | -1.89 | 0.00 | 0.00 | down |
| 45 | Teichoic acid biosynthesis | -0.60 | -1.95 | 0.00 | 0.00 | down |
| 46 | Alanine aspartate and glutamate metabolism | -0.66 | -2.00 | 0.00 | 0.00 | down |
| 47 | Two component system | -0.53 | -2.16 | 0.00 | 0.00 | down |
| 48 | *Staphylococcus aureus* infection | -0.73 | -2.52 | 0.00 | 0.00 | down |

**Table S4.** K-means clustering analysis displaying the shared DEGs with the same change pattern as the MIC value of Oxa across NC, SCV and SCV + Lon groups.

| **Gene name** | **K-means** | | |
| --- | --- | --- | --- |
|  | **NC** | **SCV** | **SCV + LON** |
| nrdF | -0.8093 | 1.2839 | -0.4746 |
| purD | -0.9029 | 1.2934 | -0.3905 |
| purL | 0.0013 | 1.1387 | -1.1400 |
| arcC | -0.6202 | 1.3295 | -0.7093 |
| purA | -0.0578 | 1.1738 | -1.1159 |
| purB | 0.0240 | 1.1366 | -1.1605 |
| purF | 0.0007 | 1.1463 | -1.1470 |
| purH | -0.5404 | 1.3014 | -0.7609 |
| purQ | 0.0507 | 1.0886 | -1.1393 |
| ymdB | 0.0691 | 1.0982 | -1.1673 |
| ndk | -0.5333 | 1.1726 | -0.6393 |
| dck | -0.4795 | 1.2709 | -0.7914 |
| purN | -0.4850 | 1.2811 | -0.7961 |
| purS | 0.1467 | 1.0368 | -1.1834 |

**Table S5.** K-means clustering analysis displaying the shared DEMs with the same change pattern as the MIC value of Oxa across NC, SCV and SCV + Lon groups.

| **Metabolite** | **K-means** | | |
| --- | --- | --- | --- |
|  | **NC** | **SCV** | **SCV + LON** |
| c-di-AMP | -0.6610 | 1.3402 | -0.6792 |

**Table S6.** MIC values of Oxa against NC, SCV and SCV + Lon groups.

|  | **NC** | **SCV** | **SCV + LON** |
| --- | --- | --- | --- |
| MIC (µg/mL) | 16 | 64 | 16 |

**Table S7.** Antibody used for immunophenotyping analysis of immune cells in bone marrow and infection-draining lymph nodes.

| Antibody | Fluorescent dye | Cat No. | Company | dilution |
| --- | --- | --- | --- | --- |
| CD45 | BV510 | 103138 | Biolegend | 1:100 |
| CD11b | FITC | 11-0112-82 | Thermo | 1:100 |
| Gr1 | PerCP-eFluor710 | 46-5931-82 | Thermo | 1:100 |
| CD3 | BUV395 | 363-0032-82 | Thermo | 1:100 |
| CD4 | FITC | 11-0042-85 | Thermo | 1:100 |
| CD8 | APC | 100712 | BioLegend | 1:100 |
| CD86 | BV421 | 105032 | BioLegend | 1:100 |
| F4/80 | APC | 17-4801-82 | Thermo | 1:100 |
| PDL1 | PE | 558091 | BD | 1:100 |
| CD206 | Biolegend | 141720 | BioLegend | 1:100 |
| PD1 | PE | 551892 | BD | 1:100 |

**Table S8.** Primers used in this study.

| **Gene** | **Upper primer sequence (5’to 3’)** | **Upper primer sequence (3’to 5’)** |
| --- | --- | --- |
| purH | GCTAAGCACACGATTGCAGG | TTTAGCAGCACCGACACGAT |
| scn | TGTGCTAGCTTGTGCCTCAT | TTGCGGGAACTTTAGCAATCG |
| hlgA | TGCAAGGCTTCATCAGCTCT | AGCATCCATGTTTCTGCCGT |
| clfA | GCGCAAAATCCAGCACAACA | GACATAACCTGCTTGGTGCG |
| VraF | GGCACAAGAAGTGTTGCGAG | GCAACAGGATCATGCGTTACC |
| blaI | CATGTCCCCTCCATACAGTTT | TGGCTGAATGGGATGTTATGAA |
| blaZ | TGCTTTAGTTTTAAGTGCATGT | TCCTTCATTACACTCTTGGC |
| mecA | TGGCAGACAAATTGGGTGGT | TGAAGCAACCATCGTTACGGA |
| purN | TGCGATTTTTGCATCAGGTTCA | ATGCTTGGCCTATTGCGTCA |
| ndk | CTGAAGGATTGGTGCTGCCA | AAGGACTAAAACTTGTCGGTGGT |
| purA | TCGGTAACGGTGTCGTTGTT | GCGCACCTTCGAAAAGTACC |
| purB | CCAGCGCGTTCGAAAATAGT | ACGCGATCGACATGCATACT |
| purF | TCCTGAAGCAGCGCAACTAA | GCATTTGGATCTACTGCGCC |
| dacA | GAAAGCGTGTGCCAAACCAA | CCAACCAGAAATTAGACGTGCG |
| gmk | TCAGGACCATCTGGAGTAGGTA | ACTTAGCTTCTACGCGCTCTC |
| relA | CTCGCTGGGGTAAATGCGTA | ACGTAGTGAACGTGAAGCGT |
| guaB | CGAAGCAGGTGTGGATGTCT | GCTAAACCAGCAGGACCCAT |
| deoD | CGTCGAAATTGCAGAAACGGT | ATGCTTCGACACCAGCGTAA |
| ALP | GCAGGCAAGACACAGACT | TGGAGGAGAGAAGGTCAGAT |
| RUNX2 | GCAGCACGCTATTAAATCCAA | GTTGGTGGCATAAAGTATGTG |
| OCN | ACCGCCTACAAACGCATCTA | AGAGGACAGGGAGGATCAAGT |

**References**

[1] B. D. Ondov, T. J. Treangen, P. Melsted, A. B. Mallonee, N. H. Bergman, S. Koren, A. M. Phillippy, *Genome biology* **2016**, 17, 132.

[2] a)H. Burhenne, V. Kaever, *Methods in molecular biology (Clifton, N.J.)* **2013**, 1016, 27; b)H. Ning, X. Liang, Y. Xie, L. Bai, W. Zhang, L. Wang, J. Kang, Y. Lu, Y. Ma, G. Bai, Y. Bai, *Frontiers in microbiology* **2022**, 13, 865045.

[3] a)C. a. L. S. I. (CLSI), **2020**; b)S. Zhang, X. Qu, H. Tang, Y. Wang, H. Yang, W. Yuan, B. Yue, *Advanced science (Weinheim, Baden-Wurttemberg, Germany)* **2021**, 8, 2100681.

[4] H. Sun, Q. Zhang, R. Wang, H. Wang, Y. T. Wong, M. Wang, Q. Hao, A. Yan, R. Y. Kao, P. L. Ho, H. Li, *Nature communications* **2020**, 11, 5263.

[5] L. F. Song, T. S. Lee, C. Zhu, D. M. York, K. M. Merz, Jr., *Journal of chemical information and modeling* **2019**, 59, 3128.

[6] a)M. Huo, L. Wang, Y. Chen, J. Shi, *Nature communications* **2017**, 8, 357; b)Y. N. Yang, S. Bernardi, H. Song, J. Zhang, M. H. Yu, J. C. Reid, E. Strounina, D. J. Searles, C. Z. Yu, *Chemistry of Materials* **2016**, 28, 704.
